# A Dual-Port, Smartphone-Linked, Pocket-Size Fluorimeter for Rapid Molecular Diagnostic Assays at Point of Care

**DOI:** 10.1109/jsen.2026.3693175

**Published:** 2026-05-21

**Authors:** Han Keun Lee, Jongwon Lim, Skye Shepherd, My Thi Tra Nguyen, Lucas Akin, Amanda Bacon, Yasmine Sidavi, Max Shurgalin, Alban Cobi, Andriy Nikolayenko, Vladimir Fuflyigin, Rashid Bashir, Xing Wang, Brian T. Cunningham

**Affiliations:** Department of Electrical and Computer Engineering, University of Illinois at Urbana-Champaign, Urbana, IL 61801 USA; Department of Bioengineering, University of Illinois at Urbana-Champaign, Urbana, IL 61801 USA; Department of Bioengineering, University of Illinois at Urbana-Champaign, Urbana, IL 61801 USA; Department of Bioengineering, University of Illinois at Urbana-Champaign, Urbana, IL 61801 USA; Department of Bioengineering, University of Illinois at Urbana-Champaign, Urbana, IL 61801 USA; Department of Bioengineering, University of Illinois at Urbana-Champaign, Urbana, IL 61801 USA; Department of Molecular and Cellular Biology, University of Illinois at Urbana-Champaign, Urbana, IL 61810 USA; GENER8 LLC, Wilmington, MA 01887 USA; Charles Stark Draper Laboratory, Cambridge, MA 02139 USA; GENER8 LLC, Wilmington, MA 01887 USA; GENER8 LLC, Wilmington, MA 01887 USA; Department of Electrical and Computer Engineering, University of Illinois at Urbana-Champaign, Urbana, IL 61801 USA; Department of Bioengineering, University of Illinois at Urbana-Champaign, Urbana, IL 61801 USA; Department of Bioengineering, University of Illinois at Urbana-Champaign, Urbana, IL 61801 USA; Department of Electrical and Computer Engineering, University of Illinois at Urbana-Champaign, Urbana, IL 61801 USA

**Keywords:** Clustered regularly interspaced short palindromic repeats (CRISPR)/Cas, molecular diagnostics, nucleic-acid amplification, point-of-care (POC) diagnostics, portable fluorimeter, recombinase polymerase amplification (RPA), reverse transcription loop-mediated isothermal amplification (RT-LAMP), target recycling amplification

## Abstract

We present the design, testing, and demonstration of a portable, pocket-size, smartphone-linked fluorimeter called the “VPodDuo.” The instrument is capable of reading the output of several fluorescence-generating biomolecular detection assays with sensitivity that is similar to larger and more expensive laboratory-based instruments. In this work, we focus on demonstrating the capability for readout of assays used to detect target nucleic acid sequences associated with infectious pathogens and cancer with incubation times of approximately 10 min. The VPodDuo features a dual-port configuration that allows simultaneous measurements of a negative experimental control (CTRL) in parallel with the test sample. We demonstrated compatibility with several assay protocols, including reverse transcription loop-mediated isothermal amplification (RT-LAMP), recombinase polymerase amplification (RPA), clustered regularly interspaced short palindromic repeats (CRISPR)/Cas, and the target recycling amplification process (TRAP). Benchmarking against three commercially available fluorimeters showed comparable detection limits for clinically relevant nucleic acid sequences, including Zika virus (ZIKV, 10^4^ copies*/μ*L), methicillin-susceptible *Staphylococcus aureus* (MSSA, 10^2^ copies*/μ*L), human immunodeficiency virus (HIV, 6.83 pM), a lung cancer-associated circulating tumor DNA sequence [L858R point-mutated epidermal growth factor receptor (EGFR) gene, 29.2 pM], and a microRNA biomarker associated with lung cancer (miR-375–3p, 500 pM). We further validated the VPodDuo’s performance under varying ambient temperatures through in-lab simulations and real-world outdoor testing using the TRAP assay for miR-375–3p detection, demonstrating cancer-associated biomarker detection at point-of-care (POC) settings. With its compact form-factor, low cost, portability, and real-time data transmission and analytical capabilities, the VPodDuo represents a promising solution for expanding access to rapid, on-site molecular diagnostics in diverse clinical and field settings.

## Graphical Abstract

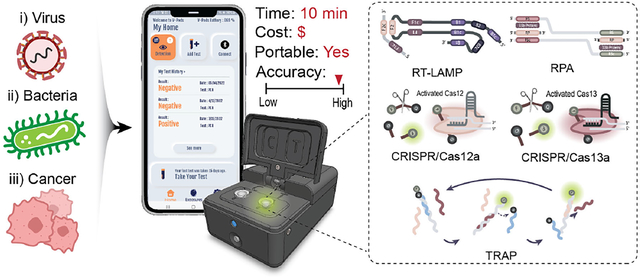

## I. Introduction

In recent years, healthcare has increasingly integrated into our daily lives. The widespread adoption of smartphones and global wireless connectivity has enabled remote healthcare, connecting patients in distant areas to medical providers for virtual consultations and monitoring of medical conditions through telehealth platforms [1]. Smart wearable devices, such as wristbands, watches, and smart patches equipped with advanced biometric sensors, have become useful tools for real-time monitoring of physiological changes, empowering individuals to take an active role in managing health [2]. In parallel, point-of-care (POC) testing has expanded access to diagnostics by enabling rapid, on-site measurements from bodily fluids without reliance on centralized laboratories [3], [4].

Despite recent advancements in POC technologies, the spectrum of detectable diseases and health conditions is highly limited, as lateral flow assays (LFAs) remain the predominant mode of detection, necessitating development of more rapid, economical, sensitive, and accurate POC testing platforms for complex medical conditions, which can include longitudinal monitoring of cancer molecular biomarkers, pathogen detection, and monitoring of chronic health conditions such as stress and cardiac disease [5]. Current commercialized POC technologies, including pregnancy tests, human immunodeficiency virus (HIV) tests, and SARS-CoV-2 antigen tests, have significantly improved accessibility to diagnostics but are constrained by critical technological limitations. Despite their portability, affordability, and ease of use, LFAs are limited by their qualitative or semiquantitative nature and inability to detect low-abundance biomarkers, making them unsuitable for early-stage detection of complex diseases and conditions or cases requiring precise quantification [6]. Additionally, their reliance upon visual interpretation of results introduces subjectivity and human error, particularly in cases where weak signals complicate the result interpretation. Lastly, because test strips are exposed during handling and readout, LFAs may be more susceptible to contamination or environmental interference in certain field workflows, which can affect result reliability.

To expand the practical scope of POCT beyond LFAs, fluorescence-based molecular assays offer a compelling path by converting low-abundance targets into strong, quantifiable signals with reduced dependence on subjective visual interpretation. Examples include isothermal nucleic-acid amplification such as reverse transcription loop-mediated isothermal amplification (RT-LAMP), recombinase polymerase amplification (RPA) [7], clustered regularly interspaced short palindromic repeats (CRISPR)/Cas-based assays that couple target recognition to reporter activation [8], and room-temperature signal-amplification chemistries such as the target recycling amplification process (TRAP) [9]. Unlike polymerase chain reaction (PCR), which requires precise thermal cycling for nucleic acid amplification, RT-LAMP operates at a constant temperature of 65 °C, simplifying both the assay workflow and supporting instrumentation, thus making it well-suited for POC deployment [10], [11], [12]. RPA, operated at a lower temperature (37 °C–42 °C), enables exponential nucleic acid amplification without thermal cycling by utilizing a recombinase complex, while significantly reducing sample-to-result time to as little as a few minutes, compared to the 1–2 h typically required for PCR [13]. CRISPR/Cas-based assays also function in a similar temperature range (37 °C–42 °C) and leverage enzymatic activation to achieve highly specific and sensitive nucleic acid detection, often capable of distinguishing sequences with a single nucleotide mismatch [14]. Lastly, TRAP further complements nucleic acid detection by utilizing a cascade of non-enzymatic chemical reactions at room temperature to amplify signals rather than nucleic acids, achieving ultra-sensitive detection at the atto-molar range [9]. Collectively, these approaches offer high sensitivity with reduced thermal requirements and represent opportunities to expand the practical scope of POC diagnostics.

Despite strong analytical potential, translation of fluorescence-based molecular assays into practical POC workflows is often limited by fluorescence readout and interpretation. In many fluorescence assays, the reading does not begin at a true zero baseline [15], [16], and initial intensity varies across runs due to reagent background, consumables/material autofluorescence, and batch-to-batch variation. Negative samples can also exhibit time-dependent background drift due to reporter behavior and nonspecific/off-target signal generation [17], [18], [19], [20], which complicates interpretation based on absolute intensity alone and reduces separation between negative and low-positive conditions. Specifically, clinically useful assays typically require low LOD [8], but detection becomes challenging when weak true-positive signals are compared to, or overwhelmed by, background signals. Moreover, nucleic acid-based chemistries are susceptible to nonspecific amplifications [19] and elevated background [20], motivating continual efforts to suppress off-target signals [21], [22], [23], [24], [25], [26], [27], [28]. For these reasons, fluorescence results are commonly interpreted relative to a matched negative control (CTRL) measured under the same run conditions, a workflow that is readily supported by laboratory fluorometers through parallelized measurement under standardized excitation and detection conditions.

This reliance on benchtop instrumentation, however, imposes cost and infrastructure burdens that limit decentralized deployment and motivates the development of miniaturized fluorescence readers. Recently reported portable quantitative instruments generally fall into two sensing architectures: imaging-based readers using camera or CMOS sensors [10], [12], [29], [30], [31], [32], [33], [34], [35], [36], [37], [38], [39], [40], [41], [42], and photodetector-based readers using photodiodes [33], [43], [44], [45], [46], [47], [48], [49]. Imaging-based readers are attractive as they provide spatial information and multiplexing capability, thus enabling simultaneous measurement of CTRL and test samples to ensure the accuracy of the performed test. However, quantitative operation often requires additional integration steps, including illumination uniformity control, optical alignment, image processing for region selection and normalization, and calibration to maintain repeatability across devices and settings, increasing the instrument complexity and integration burden. In contrast, photodiode-based readers trade spatial imaging for a simpler optical and electrical path in which output is proportional to time-integrated fluorescence, enabling a compact and manufacturable design.

Nevertheless, many photodiode-based portable readers are single-channel (single-port or single photodiode) [43], [45], [46], [47], [49], requiring sequential measurement of the test and negative CTRL and thereby increasing workflow time and susceptibility to temporal drift (instrument and/or assay kinetics). In principle, sufficiently optimized chemistry and internal quality control can reduce reliance on external CTRLs. However, established diagnostic formats, such as LFAs and many imaging-based readouts, routinely incorporate CTRL-referenced interpretation at the time of readout to verify assay integrity and reduce false positives/negatives, particularly in uncontrolled POC environments. In practice, photodiode-based instruments often underutilize CTRL-referenced interpretation because implementing dual, matched optical paths is nontrivial. It increases device complexity by requiring duplicated optics, careful alignments, detectors for each port, calibration burden, and thermal management to maintain equivalence across channels. To circumvent the complexity, for example, Lee et al. [48] developed a portable reader (CreDiT) that uses a stepping motor to rotate a disk inserted with samples and measure fluorescence sequentially, but this approach again increases system complexity, cost, and device footprint. Zhang et al. [44] developed an all-in-one microfluidic system incorporating four photodetectors for multi-color fluorescence detection [fluorescein amidite (FAM), hexachlorofluorescein (HEX), carboxy-X-rhodamine (ROX), and Cyanine 5 (Cy5)]. However, the system relies on bulky instrumentation and does not provide simultaneous negative-CTRL and test readout, limiting real-time CTRL-referenced interpretation. Together, these constraints limit the translation of highly sensitive molecular diagnostic assays into practical POC workflows, despite their strong analytical potential, which motivates the development of small, portable fluorescence readers that support simultaneous CTRL-referenced and test signal acquisition.

This article presents the development, fabrication, and performance characterization of a manufacturable, portable, pocket-sized dual-port fluorometer (Versatile-Pod-Duo; VPodDuo) that enables POC fluorescence readout with simultaneous test and negative-CTRL measurement for fluorescence-based molecular amplification assays. Building on our prior VPod system [16], which relies on additive manufacturing and supported single-sample measurement, VPodDuo is designed for manufacturability and supports concurrent measurement of two samples in standard 200 *μ*L PCR tubes, enabling matched acquisition of a test sample and a negative CTRL under the same conditions. By providing an internal reference measured in parallel, VPodDuo enables real-time comparison between test and negative-CTRL signals, facilitating prompt identification of true amplification relative to background drift and thereby reducing the effective reaction-to-answer time. A microcontroller unit (MCU) establishes a Bluetooth^1^ connection to a customized smartphone application for real-time data transfer and standardized analysis. With its compact form factor and CTRL-referenced workflow, VPodDuo is intended to reduce the infrastructure burden of fluorescence readout and facilitate decentralized molecular testing in POC environments. In this work, however, we focus on dual-port fluorescence measurement as the readout module. Assay incubation and temperature control, when required, are performed using external heating equipment and are outside the scope of the VPodDuo architecture.

To characterize VPodDuo, we performed: 1) calibration between the two photodetector channels using a high-power LED excitation source and 2) concentration-dependent fluorescence measurements using serial dilutions of FAM across multiple photodiode settings. We then compared analytical performance directly to benchtop fluorimeters to quantify agreement in sensitivity and dynamic range. We validated VPodDuo using multiple fluorescence-based molecular assays against targets, including Zika virus (ZIKV), methicillin-susceptible *Staphylococcus aureus* (MSSA), HIV, the L858R point-mutated epidermal growth factor receptor (EGFR) target for non-small cell lung cancer (NSCLC), and miR-375–3p for small cell lung cancer (SCLC). Lastly, to further validate its utility in field-deployment settings, we demonstrated the detection of miR-375–3p using room temperature TRAP assay at varying temperature conditions and confirmed the successful cancer-associated biomarker detection at POC settings. We envision that the VPodDuo system, combined with room temperature assay and dual-port configuration, can enable effective and sensitive molecular disease diagnostics at the POC, bridging current healthcare gaps by providing rapid, accessible, and cost-effective detection solutions in resource-limited settings.

## II. Development and Evaluation

### A. Workflow of VPodDuo

**Fig. 1.**
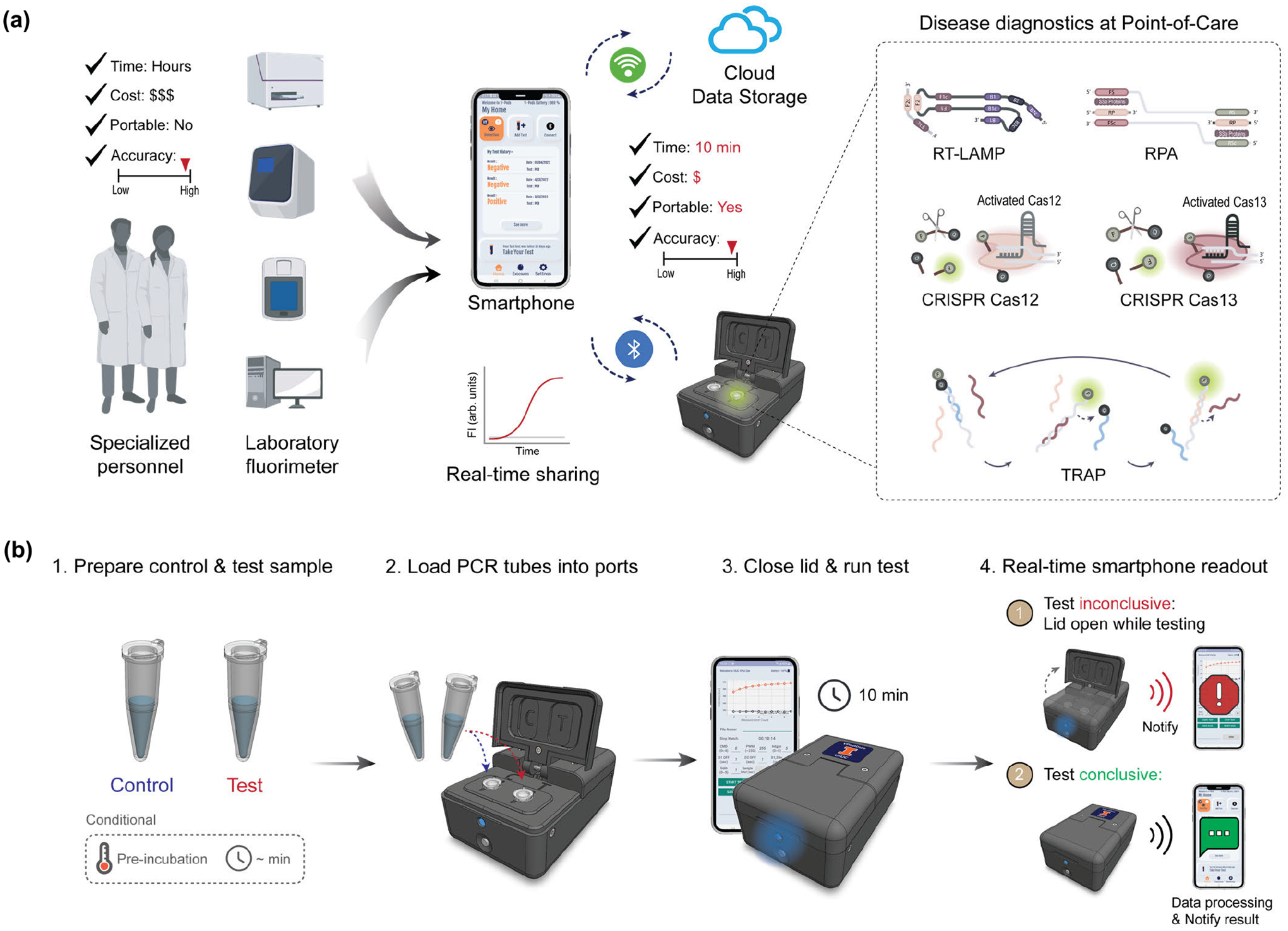
POC detection of nucleic acid biomarkers with fluorescence output using VPodDuo. (a) Schematic illustration of VPodDuo system principle and the demonstrative assays performed in this study. (b) Testing workflow of the VPodDuo system.

As illustrated in Fig. 1(a), the VPodDuo is designed as a dual-port fluorescence readout module that reduces reliance on benchtop fluorimeters and desktop-computer-based data acquisition by allowing concurrent test/CTRL measurement via Bluetooth for real-time readout and cloud-based infrastructure for data storage and sharing. The VPodDuo system is compatible with various fluorescence-based assays that utilize green fluorescence reporters, including RT-LAMP, RPA, CRISPR/Cas-based assays, and TRAP, as demonstrated in this study. To perform a test using VPodDuo, the steps illustrated in Fig. 1(b) are followed.

1. Two separate 200 *μ*L PCR tubes are prepared, representing a CTRL sample and a test sample. Depending on the assay type, pre-incubation may be required for a specific duration.
2. Once prepared, the CTRL and test samples are inserted into their respective ports.
3. The lid is closed, and the test is started via the customized smartphone app.
4. To ensure measurement accuracy, VPodDuo is equipped with a magnetic sensor to detect if the lid is open during testing, which could alter fluorescence measurements due to ambient light interference.

If an issue is detected, the system notifies the user and flags the test as inconclusive. Each test runs for 10 min, during which fluorescence measurements are continuously collected and transmitted in real-time to the customized app, where diagnostic results are processed and interpreted. The operation video of the VPodDuo system can be found in Supplementary Video 1.

### B. Device Instrumentation

**Fig. 2.**
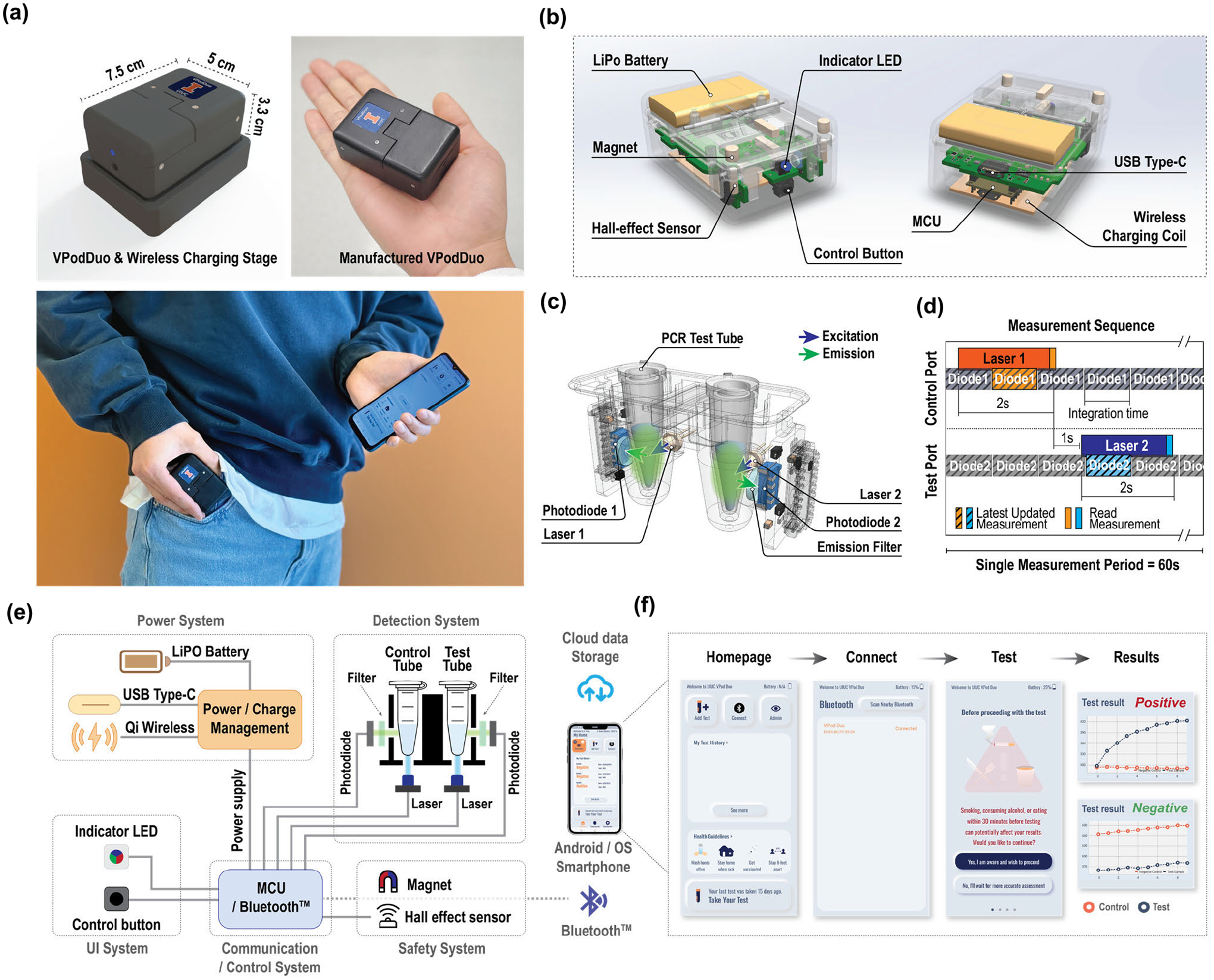
Fabrication of the pocket-sized, dual-port fluorimeter instrument. (a) External rendering and photographs. (b) Interior rendering. (c) Excitation and emission pathway of the dual-port system. (d) VPodDuo dual-port measurement sequence. (e) Schematic of the VPodDuo working principle. (f) Smartphone application for wireless connectivity and real-time result sharing.

**Fig. 3.**
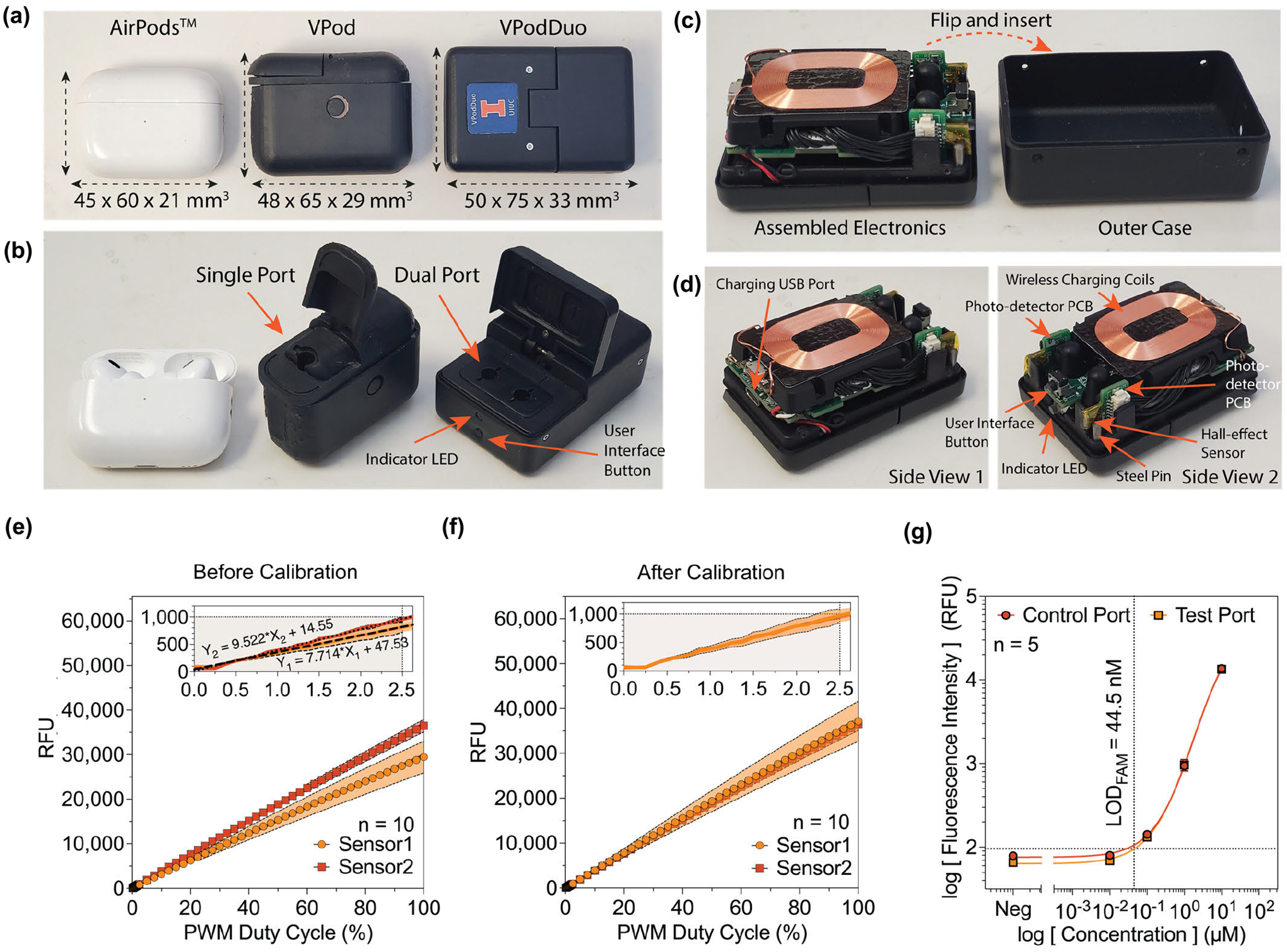
Photographs of AirPods^1^, the predecessor device (VPod), and the dual-port VPodDuo and analytical performance evaluation, and calibration. (a) Size comparison among AirPods^1^, VPod, and VPodDuo. (b) Port configuration comparison: single-port VPod versus dual-port VPodDuo. (c) Assembled electronics and outer enclosure; during assembly, the electronics module is flipped and inserted into the enclosure. (d) Side views of the assembled electronics module. (e) and (f) Intensity response of the VPodDuo to green LED light across various PWM duty cycle (%), corresponding to different FAM emission intensities, before (e) and after (f) calibration. (g) Calibrated VPodDuo response to FAM at various concentrations (10^2^, 10^1^, 100, 10^−1^, 10^−2^, and 0 *μ*M) for both CTRL (Sensor1) and test port (Sensor2). All error bars represent standard deviation (SD).

A miniaturized, low-cost, smartphone-assisted device with dual testing ports was designed with an emphasis on user-friendliness, portability, safety, and manufacturability. As illustrated in Fig. 2(a), the VPodDuo device (7.5 × 5 × 3.3 cm) fits within the palm, making it compact enough to be easily carried in a pocket. The device is equipped with a lithium-ion polymer battery and wireless charging capability, enhancing convenience and eliminating the need for external power cables, making it particularly suitable for field applications and POC diagnostics in remote or resource-limited settings. To provide real-time feedback, an indicator LED located at the front of the device [Fig. 2(b)] allows users to visually monitor the status of the device by pressing the control button, displaying information such as battery level, Bluetooth connectivity, and test progress. Furthermore, the magnetic sensor integrated into the device body detects lid closure, ensuring that tests are conducted in a well-sealed environment isolated from ambient light. This feature not only prevents fluorescence measurement inaccuracies but also enhances user safety by immediately stopping laser diode illumination when the lid is open. Since the device utilizes laser diodes to excite fluorescence reporters, this built-in safety mechanism prevents users from exposure to highly focused laser light while maintaining testing accuracy and reliability. Inside the lid are two PCR tube housing ports positioned side by side, each engraved with the letters “C” (CTRL) and “T” (test) to prevent misplacement. To facilitate ease of use by laypersons and to minimize inter-sample variation due to PCR tube orientation, each housing port is designed with an alignment groove that ensures the PCR tube can only be inserted in the correct and consistent orientation. Furthermore, the incorporated MCU enables both manual and automatic adjustment of the excitation light source and the emission light detection photodiode, supporting remote configuration and eliminating the need for unnecessary modulation by non-expert users. The electrical and optical components were selected to ensure availability from reliable vendors with sufficient capacity and quality control to support mass manufacturing. The total cost of the device components was U.S. $62.63 when only three identical instruments were prepared and hand assembled. While a comprehensive lifespan evaluation has not yet been completed, we expect multi-year functionality under standard operating conditions, provided the device is not subjected to abnormal mechanical shock, excessive humidity, or thermal stress. Supplementary Tables I and II show the bill of materials, while Supplementary Figs. 1–4 show the electrical schematics. A detailed exploded diagram of the device is shown in Supplementary Fig. 5. The photographs of the assembled device and its comparison to its predecessor VPod and Apple AirPods are shown in Fig. 3(a)–(d).

### C. Excitation and Emission Collection Sequence

Fig. 2(c) illustrates the excitation and emission collection mechanism of the dual-port configured VPodDuo system. Each port consists of its own photodiode and laser diode pair, where the laser diode illuminates the sample from the side, parallel to the ground, while the emitted fluorescence is collected by a low-cost, highly sensitive photodiode sensor (VEML7700, Vishay Semiconductors), positioned orthogonal to laser (ams OSRAM; 450 nm; 110 mW) illumination. A 510 nm long-pass filter (Chroma) is placed in front of the sensor to selectively capture fluorescence emission while blocking wavelengths from the excitation source. The excitation and emission measurement sequence is depicted in Fig. 2(d). The two ports are operated in a time-multiplexed manner such that only one laser is active at a time. For a given port, the laser is turned on, and the corresponding photodiode is sampled programmatically approximately 2 s after illumination begins. After acquisition from the first port is completed, the system switches to the second port and repeats the same sequence (laser activation followed by photodiode sampling). A detailed explanation of this programmatic sequence is described in Supplementary Method S1.

### D. User App Interface and Device Operation

Detailed screenshots of the application interface and a demonstration video can be found in Supplementary Figs. 6 and 7, and Supplementary Video 1, respectively. Fig. 2(e) and (f) depicts overall device functionality, where an (MCU, Arduino Nano BLE 33) serves as the communication and control hub, enabling connectivity to a customized smartphone application. To initiate Bluetooth pairing, the user presses the control button for approximately three seconds, causing the LED indicator to blink white, signaling that the device is in discoverable mode. The application then scans for available Bluetooth devices nearby, and upon successful connection, the LED indicator turns solid blue, confirming the established link. Once the test begins, the LED indicator blinks blue, indicating that the fluorescence measurement process is active. Upon test completion, the application presents the final diagnostic results, along with a graphical representation of the raw fluorescence measurements for further analysis. For user-friendliness, the device also features wireless charging capability and connectivity to cloud-based data storage. The smartphone application was developed using Java and Kotlin programming language, while the MCU was programmed with C++ programming language.

### E. Calibration of Dual-Port Sensors

To ensure diagnostic reliability, each port of the VPodDuo system must be properly calibrated to maintain consistent and accurate fluorescence measurement. Ideally, the fluorescence reading of a CTRL sample from the CTRL port should be within a similar range as that from the test port. Inconsistencies between the two ports could introduce systematic errors, potentially compromising diagnostic accuracy. For instance, if the fluorescence reading of a CTRL sample from the CTRL port is significantly higher than that from the test port, the detection limit of the system is impaired, increasing the potential for false-negative detection. Conversely, if the fluorescence reading of a CTRL sample is significantly lower than that from the test port, it could lead to false-positive results, as the system could incorrectly interpret any signal from the test port as a meaningful photodiode output. To mitigate these issues, rigorous characterization and calibration are performed to ensure that both ports produce comparable fluorescence readings. Precise calibration is especially important when measuring subtle changes in fluorescent intensity and quantitation of small increases of fluorescence intensity above the zero-intensity background.

A total of three VPodDuo identical instruments were fabricated by a medical device manufacturer (Gener8 LLC). To initially assess the analytical performance of the instruments, a FAM reporter with an initial concentration of 100 *μ*M was serially diluted to obtain final concentrations of 100, 10, 1, 0.1, 0.01, and 0 *μ*M in a 100 *μ*L volume. During the experiments, fluorescence readings for each concentration were taken at various integration times of 100, 400, and 800 ms, while the sensor analog gain was fixed at × 1. Additionally, the excitation power from the laser was measured and set to 10 mW via pulsewidth modulation (PWM) [Supplementary Fig. 8(a)]. The experimental results from all three instruments are presented in Supplementary Fig. 8(b). The three instruments demonstrated strong concentration-dependent responses. Fluorescence measurements taken with an 800 ms integration time exhibited signal saturation at 100 *μ*M, limiting the dynamic range. In contrast, measurements at 100 ms integration time did not saturate but were limited by sensitivity. Considering both sensitivity and dynamic range, an integration time of 400 ms was selected for all subsequent experiments.

A discrepancy in fluorescence measurements between the two ports was observed at low concentrations of the FAM reporter across all three instruments. To address this issue, a calibration method was developed using a high-power LED source (RGBW LED, Chanzon) to mimic the emission response of the FAM reporter. Supplementary Fig. 9(a) presents an image of the fabricated calibration module, where the green LED intensity is precisely controlled via PWM; the PWM value was set digitally with 12-bit resolution (0–4095). For plotting, the PWM value was converted to PWM duty cycle (%) as 100 × (PWM value*/*4095). As illustrated in Supplementary Fig. 9(b), the green light is illuminated from the top, maintaining identical experimental conditions as in the fluorescence measurement setup, except for the absence of FAM reporters. To validate the suitability of the LED source for calibration, the spectral responses of the red, green, blue, and white light from the LED were measured and compared to the emission spectrum of the excited FAM reporter [Supplementary Fig. 9(c)]. The spectral response of the green LED is closely aligned with the FAM emission spectrum, confirming its suitability as a calibration reference. Fig. 3(e) and Supplementary Fig. 9(d) present the experimental results, where LED-driven reference light measurements for both ports were taken without a PCR tube while sweeping the PWM duty cycle (%). The test port (sensor2) exhibited greater sensitivity compared to the CTRL port (sensor1), while the CTRL port showed greater variability between samples. To mitigate this issue, calibration was performed by carefully selecting an intensity response window where a linear fitting minimized the residual difference between the measured values and the fit linear model [Supplementary Fig. 9(e)]. The obtained linear equations were then solved by letting *X*_1_ = *X*_2_ and solving for *Y*_2_ to convert the CTRL port response (*Y*_1_) to the scale of the test port (*Y*_2_). The calibration results are presented in Fig. 3(f) and Supplementary Fig. 9(f), confirming the effectiveness of the calibration process in reducing port-to-port variability.

### F. Port Isolation and Long-Term Stability Characterization

Further characterization was performed to evaluate dark current and operational longevity [Supplementary Fig. 9(g) and (h)]. The dark current remained at zero for both sensors, confirming complete enclosure of both ports and effective isolation from ambient light. The laser power for both sensors remained stable over an approximately 38-h duration, independent of battery level, demonstrating consistent performance. This translates to a testing capacity exceeding 230 tests per charge, ensuring reliable long-term operation.

Port-to-port optical isolation was also evaluated using the excitation/detection scheme illustrated in Supplementary Fig. 10. We compared two operating conditions. In the in-phase condition, each port was measured using its own excitation and detector (i.e., Port 1 excitation with Photodiode 1 readout, and Port 2 excitation with Photodiode 2 readout), which captures the expected within-port excitation background. In the out-of-phase condition, excitation from one port was applied while the detector on the opposite port was sampled (i.e., Port 1 excitation with Photodiode 2 readout, and Port 2 excitation with Photodiode 1 readout), which directly tests inter-port optical crosstalk. As expected, non-zero background levels were observed in the uncalibrated, in-phase condition [Supplementary Fig. 10(b)] due to within-port scatter and minor port-to-port differences in detector responsivity and optical alignment/filter transmission. In contrast, the out-of-phase measurements remained at zero, indicating no detectable inter-port optical crosstalk and confirming independent operation of the two ports under the measurement modes [Supplementary Fig. 10(c)–(h)].

### G. Analytical Performance Evaluation

After calibrating the system using a high-power LED, the fluorescence intensity of serially diluted FAM concentrations was measured using VPodDuo to confirm the close alignment between the CTRL and test ports in terms of signal response. The detection limit was calculated and directly compared with other lab-grade fluorimeters. Fig. 3(g) presents the calibrated concentration-dependent response of the VPodDuo device, while Supplementary Fig. 11(a)–(c) displays measurements gathered with commercially available, laboratory-based fluorimeters, namely, the Qubit^1^, QuantStudio^1^, and a fluorescence microplate reader (Molecular Devices, CA). The limit of detection (LOD_FAM_) for the VPodDuo device was determined to be 44.5 nM, while the LOD_FAM_ for the laboratory fluorimeters was 0.5, 16.6, and 5 nM, respectively. Despite the approximately two order-of-magnitude decrease in detection limit compared to Qubit^1^, the experiment results showed comparable analytical performance to the other two laboratory fluorimeters, highlighting the strong detection capacity of an inexpensive and palm-sized device. Moreover, it is important to note that the commercial fluorimeters (e.g., Qubit^1^) typically utilize a single excitation light source and a single photodetector, which minimizes measurement variability arising from multiple excitation and detection channels that directly influence the detection limits of the instrument. In contrast, the VPodDuo employs multiple optical sources to enable simultaneous detection of test and CTRL samples to minimize the testing time while achieving high analytical sensitivity. Supplementary Fig. 12 illustrates the effect of using multiple optical sources on the detection limit. When each port (CTRL and test) was analyzed independently using a dedicated excitation source and photodiode, the LOD_FAM_ were 14 and 24.8 nM, respectively. However, when the CTRL port served as a negative reference for the test port without applying calibration, the LOD_FAM_ increased substantially to 128 nM. Only after applying proper calibration did the LOD_FAM_ improve to 44.5 nM, underscoring both the challenge of achieving low detection limits with multiple sources and the critical importance of calibration. Despite this more complex configuration, the VPodDuo achieves comparable analytical performance, demonstrating the robustness and precision of its design.

### H. Hall Effect Sensor Integration

The VPodDuo device incorporates a hardware interlock to prevent laser operation when the enclosure lid is open. A low-power omnipolar Hall-effect switch (DRV5032AJDMRR) is used as a magnetic lid-closed detector and provides an open-drain digital output. When the lid is open (magnet absent), the DRV5032 output is high impedance, and a pull-up resistor drives the gate of an nMOS transistor (SiA938DJT) high, turning it on and clamping the PWM CTRL node to ground. This forces the CTRL input of the boost LED driver (TPS61043) low, thereby disabling PWM modulation and preventing laser emission (Supplementary Figs. 3 and 4). When the lid is closed (magnet present), the DRV5032 actively pulls its output low, pulling the nMOS gate low and turning the clamp transistor off. The PWM signal can then reach the TPS61043 CTRL pin to regulate the laser drive.

The magnetic flux density along the magnet axis can be approximated as a function of the air-gap distance *g* by

(1) $$B(g)=\frac{B_{r}}{2}\left[\frac{g+L}{\sqrt{{\left(g+L\right)}^{2}+R^{2}}}-\frac{g}{\sqrt{g^{2}+R^{2}}}\right]$$

where *B*_*r*_ is the remanent flux density of the permanent magnet, *g* is the separation between the magnet face and the Hall sensing element, *L* is the magnet thickness, and *R* is the magnet radius. The hall effect sensor pulls the node low when the local flux density magnitude exceeds the device operating threshold (*B*_*threshold*_). Otherwise, the output becomes high impedance, and the node is pulled high by the external pull-up resistor. Empirically, a separation of approximately 12 mm was required for reliable magnet detection in the magnet-only configuration [Supplementary Fig. 13(a)]. To increase the actuation distance within the device’s small footprint, a steel dowel pin was positioned adjacent to the Hall switch, and a disk magnet was embedded in the lid [Supplementary Fig. 13(b) and (c)]. Upon lid closure, the magnet couples to the steel pin, which concentrates magnetic flux near the sensor, increasing the effective field at the Hall element and extending the detectable separation to approximately 15 mm. The resulting digital lid-state signal is used by the mobile application to notify the user and stop the laser illumination [Supplementary Fig. 13(d)].

## III. Experimental Results for Molecular Biomarker Detection

### A. RT-LAMP Assay for ZIKVs Detection

ZIKV is a flavivirus known to cause fetal microcephaly, a severe developmental disorder in newborn babies, resulting from maternal infection during early pregnancy. The primary mode of transmission is through the bites of infected *Aedes aegypti* mosquitoes. Initially considered a minor public health concern, ZIKV quickly spread across South America, prompting the World Health Organization (WHO) to declare a global health emergency in early 2016 [50]. Although reported infections have declined in recent years, women of reproductive age in Latin America and the Caribbean still remain at risk, underscoring the ongoing need for accessible and reliable diagnostic tools [51].

To demonstrate the capability for ZIKV detection at POC using the VPodDuo system, an RT-LAMP assay was employed, and the experimental results were compared with those from the QuantStudio^1^ fluorimeter. The working principle of the RT-LAMP assay is illustrated in Supplementary Fig. 14(a). Briefly, RT-LAMP achieves nucleic acid amplification via reverse transcriptase, Bst DNA polymerase and six primers that target distinct regions of the ZIKV RNA sequence. The reaction is initiated by the forward internal primer (FIP) and backward internal primer (BIP), which anneal to complementary regions of the target RNA. Reverse transcriptase then converts the RNA into cDNA, followed by strand synthesis by Bst DNA polymerase. The Forward Outer Primer (F3) and Backward Outer Primer (B3) then bind to outer regions of the newly synthesized strand, causing strand displacement and releasing a single-stranded DNA intermediate. This displaced strand undergoes self-annealing at its complementary ends, forming a looped dumbbell structure, which serves as a self-priming template for further amplification [10]. To generate fluorescence readout, we utilized EvaGreen, a double-stranded DNA (dsDNA) intercalating dye. As the amplification reaction proceeds and the intercalating molecules themselves are inserted between the base pairs of the DNA double helix, the accumulation of dsDNA leads to an increase in fluorescence intensity proportional to the amount of amplicon generated.

**Fig. 4.**
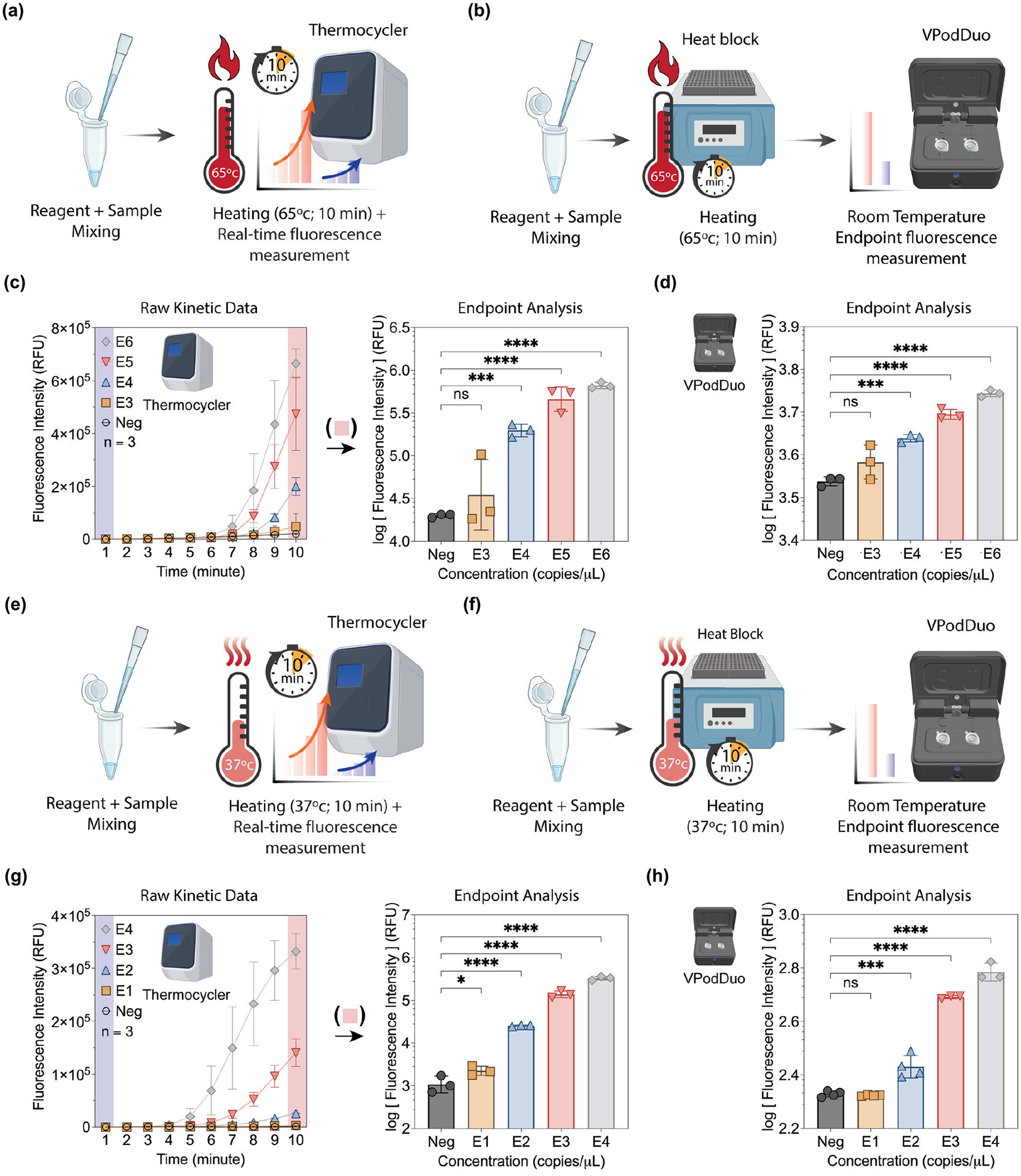
Detection of ZIKV and MSSA using RT-LAMP and RPA, respectively, on the VPodDuo system and QuantStudio^1^. (a) Experimental workflow for RT-LAMP performed on the thermocycler (QuantStudio^1^), where samples were incubated at 65 °C for 10 min, during which the fluorescence signals were measured at 1 min intervals. (b) Experimental workflow for RT-LAMP performed on VPodDuo, where samples were incubated at 65 °C for 10 min, followed by a single fluorescence measurement. (c) RT-LAMP fluorescence measurements from QuantStudio^1^, shown as a kinetic graph and an endpoint analysis graph (fluorescence at *t* = 10 min) for target concentrations of 10^6^, 10^5^, 10^4^, 10^3^, and 0 copies*/μ*L. Only the endpoint analysis was used since VPodDuo does not provide the kinetic response due to the need for a separate heat incubation. ANOVA p-values for the respective concentrations are <0.0001, <0.0001, 0.0004, and 0.4204. (d) RT-LAMP fluorescence measurements from the VPodDuo system, recorded after 10 min of heat incubation at 65 °C (kinetic graph not applicable). ANOVA test p-values for the respective concentrations are <0.0001, <0.0001, 0.0003, and 0.0609. (e) Experimental workflow for RPA performed on QuantStudio^1^ where samples were incubated at 37 °C for 10 min, during which the fluorescence signals were measured at 1 min intervals. (f) Experimental workflow for RPA performed on VPodDuo, where samples were incubated at 37 °C for 10 min, followed by a single fluorescence measurement. (g) RPA fluorescence measured on QuantStudio^1^, shown as a kinetic graph and an endpoint analysis graph for target concentrations of 10^4^, 10^3^, 10^2^, 10^1^, and 0 copies*/μ*L. ANOVA p-values for the respective concentrations are <0.0001, <0.0001, <0.0001, and 0.0149. (h) RPA fluorescence measured on VPodDuo system after 10 min incubation at 37 °C (kinetic graph not applicable). One-way ANOVA p-values for the respective concentrations are <0.0001, <0.0001, 0.0002, and 0.9932. All error bars represent standard deviations (SD).

To evaluate the analytical performance of VPodDuo, gamma-irradiated and inactivated ZIKV (PRVABC59, NR-50547, BEI Resources) was spiked into the buffer and serially diluted to 10^6^, 10^5^, 10^4^, 10^3^, and 0 copies*/μ*L in a final 16 *μ*L reaction volume. To enable direct comparison, the prepared samples were analyzed using QuantStudio^1^ according to the standard RT-LAMP assay procedure, including the incubation at 65 °C [Fig. 4(a)] and real-time kinetic measurement. The reaction tubes were then removed from the thermocycler, and immediately measured using VPodDuo for single, endpoint fluorescence readout [Fig. 4(b)]. Each concentration test was conducted in triplicate. The CTRL and test samples were loaded into their corresponding ports, enabling real-time side-by-side measurement, eliminating the need for separate CTRL testing. The experimental results showed that the VPodDuo system achieved an LOD of 10^4^ copies*/μ*L, which was consistent with the LOD tested by QuantStudio^1^ [Fig. 4(c) and (d)]. The detailed assay compositions are described in Supplementary Methods S2.

### B. RPA Assay for MSSA Detection

MSSA is a bacterial pathogen responsible for a wide range of infections, including pneumonia, endocarditis, osteomyelitis, and postsurgical wound infections. While MSSA remains susceptible to *β*-lactam antibiotics, rapid and accurate detection is essential for early intervention and appropriate antibiotic selection, minimizing unnecessary use of broad-spectrum antibiotics and mitigating the risk of antimicrobial resistance (AMR) [52].

To enable POC detection of MSSA, the RPA assay was employed, leveraging the VPodDuo system for fluorescence-based readout. As illustrated in Supplementary Fig. 14(b), RPA achieves nucleic acid amplification primarily through the coordinated action of a recombinase complex, forward and backward primers (FP, BP), and DNA polymerase. Briefly, the recombinase complexes, initially formed by binding to the FP and BP, facilitate the annealing of these primers to homologous sequences within the dsDNA template. Single-stranded binding proteins then stabilize the displaced DNA strand. Once the recombinase complexes disassemble, DNA polymerase extends the primers, leading to cyclic amplification and exponential DNA replication [7]. For fluorescence generation, we employed a fluorogenic Exo probe, which contains a fluorophore (F) and a quencher (Q) separated by an abasic (tetrahydrofuran, THF) residue. Upon specific hybridization to the target DNA (during and after the amplification process), exonuclease III recognizes the THF site and cleaves the probe. This enzymatic cleavage physically separates the fluorophore from the quencher, resulting in an increase in fluorescence signal.

Serially diluted genomic DNA from the MSSA strain Seattle 1945 (25923D-5, ATCC) templates in buffer at concentrations of 10^4^, 10^3^, 10^2^, 10^1^, and 0 copies*/μ*L in a final 50 *μ*L reaction volume were tested on both the VPodDuo system and QuantStudio^1^. To enable direct comparison, the prepared samples were analyzed using QuantStudio^1^ according to the standard assay procedure, including the incubation at 37 °C [Fig. 4(e)] and real-time kinetic measurements. The reaction tubes were then removed from the thermocycler and immediately measured using VPodDuo for single, endpoint fluorescence readout at room temperature, conducted in triplicate [Fig. 4(f)]. The experimental results demonstrated an LOD of approximately 10^2^ copies*/μ*L for the VPodDuo system, whereas the LOD for QuantStudio^1^ was approximately 10^1^ copies*/μ*L, indicating a one-order-of-magnitude difference in the limit of detection [Fig. 4(g) and (h)]. This discrepancy is expected as QuantStudio^1^ exhibits approximately twice the sensitivity in LOD when detecting FAM reporters. Despite this reduction in sensitivity, the portability, accessibility, and cost-effectiveness of the VPodDuo system make it a highly viable diagnostic tool, well-suited for POC applications where rapid and decentralized MSSA detection is needed. The detailed assay compositions are described in Supplementary Methods S3.

### C. CRISPR/Cas13a Assay for HIV Detection

Since its first identification in 1983, HIV has had a profound impact globally, exceeding expectations in both its reach and severity. According to the WHO, an estimated 39.9 million people worldwide were living with HIV by 2023, with approximately 1.3 million new infections occurring annually [53]. Although new infections have been declining, achieving the UNAIDS target of reducing annual new HIV cases to below 370 000 by 2025 remains a significant challenge due to current technological limitations in early HIV detection. A recent study has raised concerns about the effectiveness of POC HIV testing, particularly LFA-based tests, which have shown 100% failure in detecting acute HIV infections – the earliest stage of HIV infection where the infectivity is at its highest [54]. Given that early detection is crucial for timely intervention and transmission prevention, reliance on conventional POC LFA-based tests presents a critical diagnostic gap. As a result, molecular laboratory testing remains vital, highlighting the urgent need for rapid, accurate, and highly sensitive molecular POC diagnostic tools that can effectively detect HIV at early stages.

To address this, the molecular detection of HIV was demonstrated using the VPodDuo system, leveraging a CRISPR/Cas13a-based assay. The working principle of the CRISPR/Cas13a assay is illustrated in Supplementary Fig. 14(c). Briefly, *Leptotrichia wadei* Cas13a (LwaCas13a) can be programmed to target a specific nucleic acid sequence through the utilization of the guide RNA (gRNA) with a sequence complementary to the target HIV RNA sequence. Upon binding to the gRNA, Cas13a forms a ribonucleoprotein (RNP) complex that recognizes the target HIV sequence through partial complementary pairing between the gRNA spacer and the target sequence. In the presence of the target, the complex binds to the complementary region, triggering Cas13a activation, a state where it exhibits collateral single-stranded RNA (ssRNA) cleavage (i.e., trans-cleavage). The activated enzyme indiscriminately cleaves nearby fluorophore-quencher (FQ, Alexa Fluor 488 and 3’ Iowa Black) reporter probes to disrupt their proximity-based quenching interaction, resulting in fluorescence emission [8]. The VPodDuo system captures this fluorescence signal, enabling real-time molecular HIV detection at POC.

**Fig. 5.**
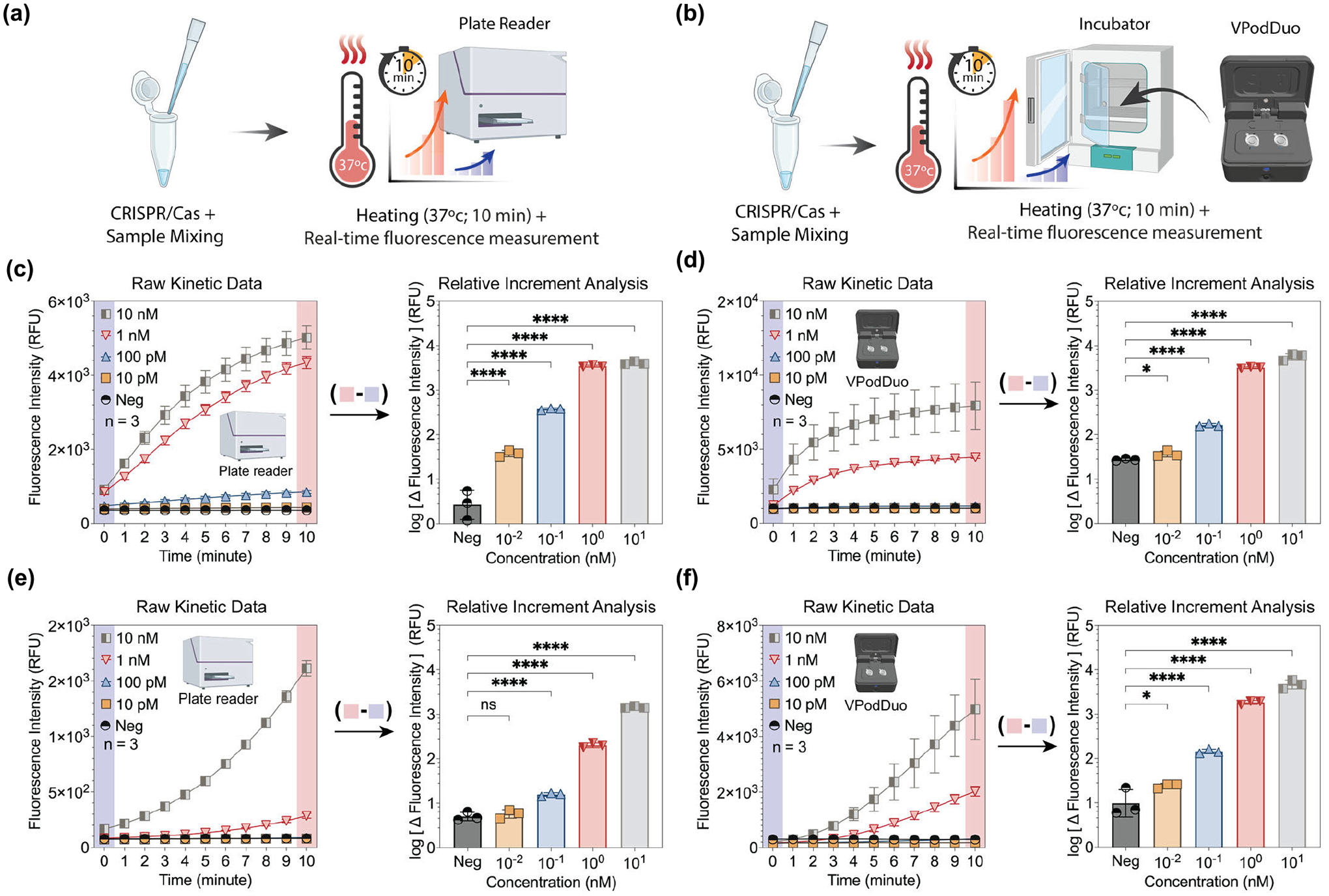
Detection of HIV and L858R point-mutated EGFR gene using CRISPR/Cas-based assays on the VPodDuo system and a laboratory microplate reader. (a) Experimental workflow for CRISPR/Cas13a (HIV) and CRISPR/Cas12a (EGFR) assays performed on the microplate reader, where samples were incubated at 37 °C for 10 min, during which fluorescence signals were measured at 1 min intervals. (b) Experimental workflow for the same assays performed on VPodDuo, where samples were incubated at 37 °C for 10 min, during which fluorescence signals were measured at 1 min intervals. (c)–(f) Fluorescence data are presented as log-scaled kinetic traces with a corresponding relative signal increment (Δ *fluorescence*) computed as *F*_10 min_–*F*_0 min_. (c) and (d) HIV detection results for the microplate reader and VPodDuo, respectively. For the microplate reader, one-way ANOVA p-values for target concentrations of 10^1^, 10^0^, 10^−1^, and 10^−2^ nM were <0.0001 for all concentrations. For VPodDuo, the corresponding p-values were <0.0001, <0.0001, <0.0001, and 0.0313, respectively. (e)–(f) L858R EGFR detection results for the microplate reader and VPodDuo, respectively. For the microplate reader, one-way ANOVA p-values for 10^1^, 10^0^, 10^−1^, and 10^−2^ nM were <0.0001, <0.0001, <0.0001, and 0.6912, respectively. For VPodDuo, the corresponding p-values were <0.0001, <0.0001, <0.0001, and 0.0287, respectively. All error bars represent SD.

Experiments were conducted using target concentrations of 10^1^, 10^0^, 10^−1^, 10^−2^, and 0 nM, prepared by serial dilution in buffer to a final reaction volume of 100 *μ*L. First, samples were measured using a plate reader at 37 °C for 10 min, during which fluorescence was recorded in real-time at 1 min intervals [Fig. 5(a)]. For direct comparison, VPodDuo was placed inside an incubator maintained at 37 °C to match the same temperature condition [Fig. 5(b)], and the same concentrations were then tested, with each concentration measured in triplicate. The LOD for the VPodDuo system was determined to be approximately 6.83 pM, while the LOD for the laboratory plate reader was approximately 7.74 pM [Fig. 5(c) and (d)]. Despite being small and inexpensive, VPodDuo showed comparable LOD when compared to the laboratory-based plate reader. The detailed assay composition is described in Supplementary Methods S4.

### D. CRISPR/Cas12a Assay for L858R Point-Mutated EGFR Gene Detection

The EGFR gene has been extensively studied as a key biomarker for oncology research due to its critical role in regulating cell growth and proliferation. Specifically, mutations in EGFR—particularly in Exon 19 deletions and Exon 21 L858R point mutations—are known to drive tumorigenesis, a process which causes normal cells to transform into cancerous cells through uncontrolled signaling and proliferation [55]. Moreover, a growing body of studies has established a strong correlation between the onset of NSCLC and the L858R point-mutated EGFR gene, underscoring its clinical significance [56]. Thus, monitoring L858R mutation levels is particularly of importance for the early NSCLC diagnosis, enabling timely intervention and improved patient outcomes.

To demonstrate POC detection of the L858R point-mutated EGFR gene, we employed a CRISPR/Cas12a-based assay using the VPodDuo system. The working principle remains identical to the CRISPR/Cas13-based assay for HIV detection, except that Cas12a specifically targets and cleaves ssDNA instead of ssRNA. Targets were serially diluted in buffer to 10^1^, 10^0^, 10^−1^, 10^−2^, and 0 nM in a 100 *μ*L total reaction volume. Samples were first measured on a plate reader at 37 °C for 10 min with fluorescence recorded at 1 min intervals [Fig. 5(a)]. For direct comparison, VPodDuo was placed in a 37 °C incubator to match the same temperature condition [Fig. 5(b)], and the same concentrations were tested in triplicate. The LOD for the VPodDuo system was found to be approximately 29.2 pM, while the LOD for the laboratory microplate reader was approximately 47 pM, demonstrating comparable performance despite the VPodDuo being compact and portable [Fig. 5(e) and (f)]. The detailed assay composition is described in Supplementary Methods S5.

### E. TRAP Assay for miR-375–3p Detection

MiRNAs are short, non-coding RNA molecules that regulate gene expression by binding to specific sequences on target messenger RNAs (mRNAs), resulting in either mRNA degradation or inhibition of protein translation. Since their discovery, a growing body of evidence has established their role as key regulators of cancer cell proliferation, apoptosis, and migration, opening the pathway for miRNA expression to serve as a reliable biomarker for cancer diagnosis. Among these, miR-375 has been identified as a critical modulator in multiple cancers, including SCLC. Although miR-375 is known to play a tumor-suppressive role in multiple different cancers, aberrant upregulation of miR-375–3p has been strongly correlated with increased tumor growth in the case of SCLC [57]. Thus, there is an ongoing critical need to develop rapid and accessible detection methods for miR-375–3p to enable early SCLC diagnosis.

To address this need, the TRAP assay was utilized in conjunction with the VPodDuo system to demonstrate POC detection of miR-375–3p. Unlike other assays that require heat incubation to initiate enzymatic nucleic acid amplification, TRAP operates via a room-temperature chemical signal amplification reaction, making it highly suitable for sensitive and rapid molecular diagnostics in POC settings. The working principle of TRAP is illustrated in Supplementary Fig. 14(d). The TRAP process relies on branch migration displacement reactions that are initiated through the binding of short regions called toeholds. These toeholds allow an invading strand to displace a protecting strand from a duplex in an entropy-driven enzyme-free process. Briefly, TRAP is governed by these toehold-mediated strand displacement reactions, where a linker strand with a FAM fluorophore at its end and a protector strand with a black hole quencher (BHQ) at its end are self-annealed to form an initial quenched complex. Upon introducing the target miR-375–3p, the toehold region of the linker strand facilitates strand displacement, leading to the release of the protector strand and unquenching the FAM fluorophore. Subsequently, this exposes a second toehold region that is originally sequestered, allowing the target-linker duplex to undergo a secondary strand displacement initiated by a fuel strand (probe), which releases the target miRNA for further recycling. This enables continuous, linear signal amplification without the need for enzymatic reactions [9].

**Fig. 6.**
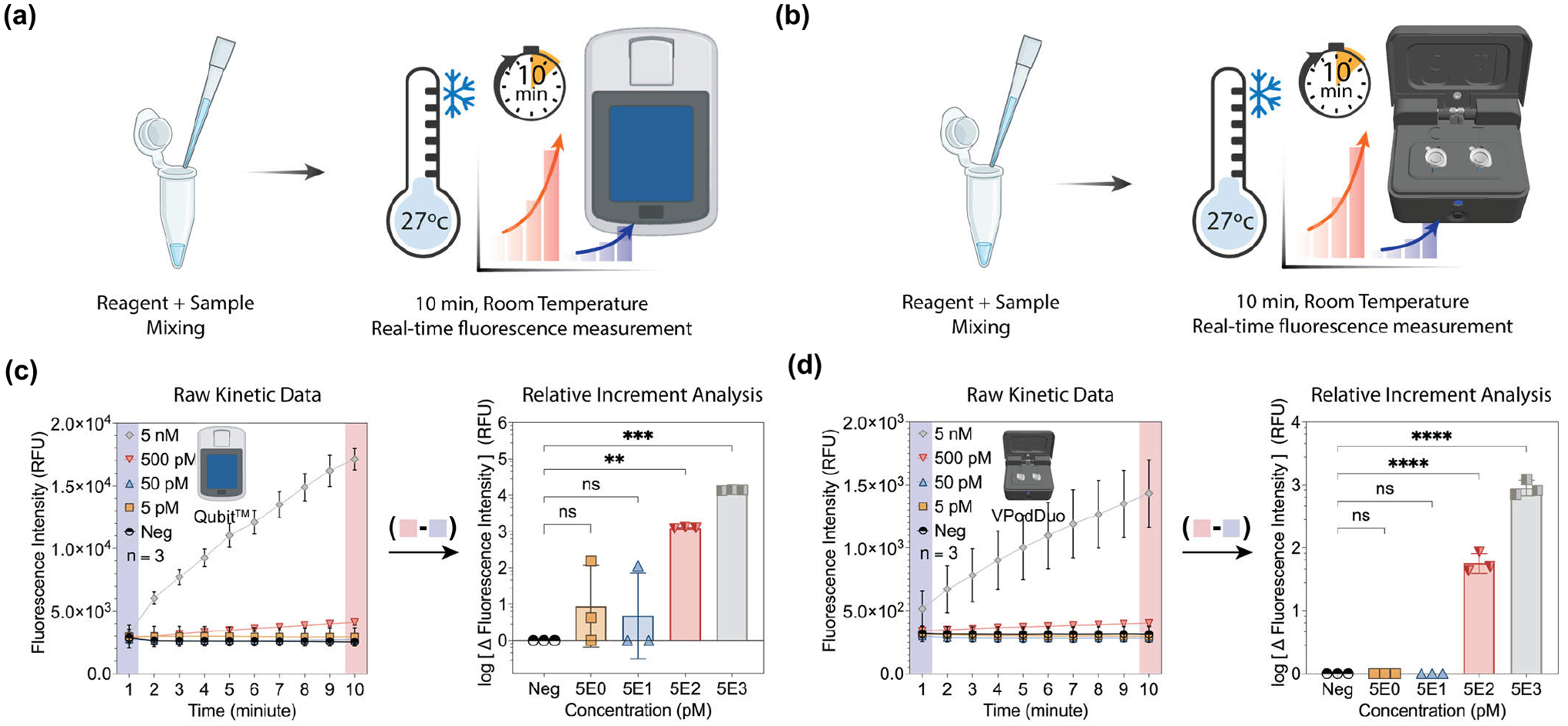
Detection of miR-375–3p using TRAP assays on the VPodDuo system and Qubit^1^. (a) Experimental workflow for TRAP performed on Qubit^1^, where samples were incubated at room temperature for 10 min, during which the fluorescence signals were measured at 1 min intervals. (b) Experimental workflow for TRAP performed on VPodDuo, where samples were incubated at room temperature for 10 min, during which the fluorescence signals were measured at 1 min intervals. (c) and (d) Fluorescence measurements from Qubit^1^ (c) and VPodDuo (d) are shown as a kinetic graph with a corresponding relative signal increment (Δ fluorescence) computed as *F*_10 min_–*F*_0 min_ for target concentrations of 5 × 10^3^, 5 × 10^2^, 5 × 10^1^, 5 × 10^0^, and 0 pM. One-way ANOVA p-values for Qubit^1^ were 0.0001, 0.0014, 0.6265, and 0.3763 for respective concentrations. The corresponding p-values for VPodDuo were <0.0001, <0.0001, >0.9999, and >0.9999, respectively. All experiments were conducted at room temperature. Error bars represent the SD.

The detection of miR-375–3p was demonstrated using TRAP in conjunction with both Qubit^1^ and VPodDuo systems. The relative fluorescence increment values were recorded at room temperature without any heat incubation, and the results were directly compared [Fig. 6(a)]. The experimental conditions for VPodDuo measurements remained identical to those of the Qubit^1^ [Fig. 6(b)]. The initial target stock was serially diluted with buffer to final concentrations of 5000, 500, 50, 5, and 0 pM in a final 100 *μ*L reaction volume. The prepared samples were then loaded into their corresponding ports, and fluorescence measurements were recorded at 1 min intervals for 10 min, generating a kinetic fluorescence profile over time. The experimental results indicated that both the VPodDuo and Qubit^1^ achieved an LOD of approximately 500 pM, demonstrating comparable sensitivity [Fig. 6(c) and (d)]. The detailed assay compositions are described in Supplementary Methods S6. Table I summarizes the detection results.

### F. miR-375–3p Detection With TRAP at Field Condition Environment

**Fig. 7.**
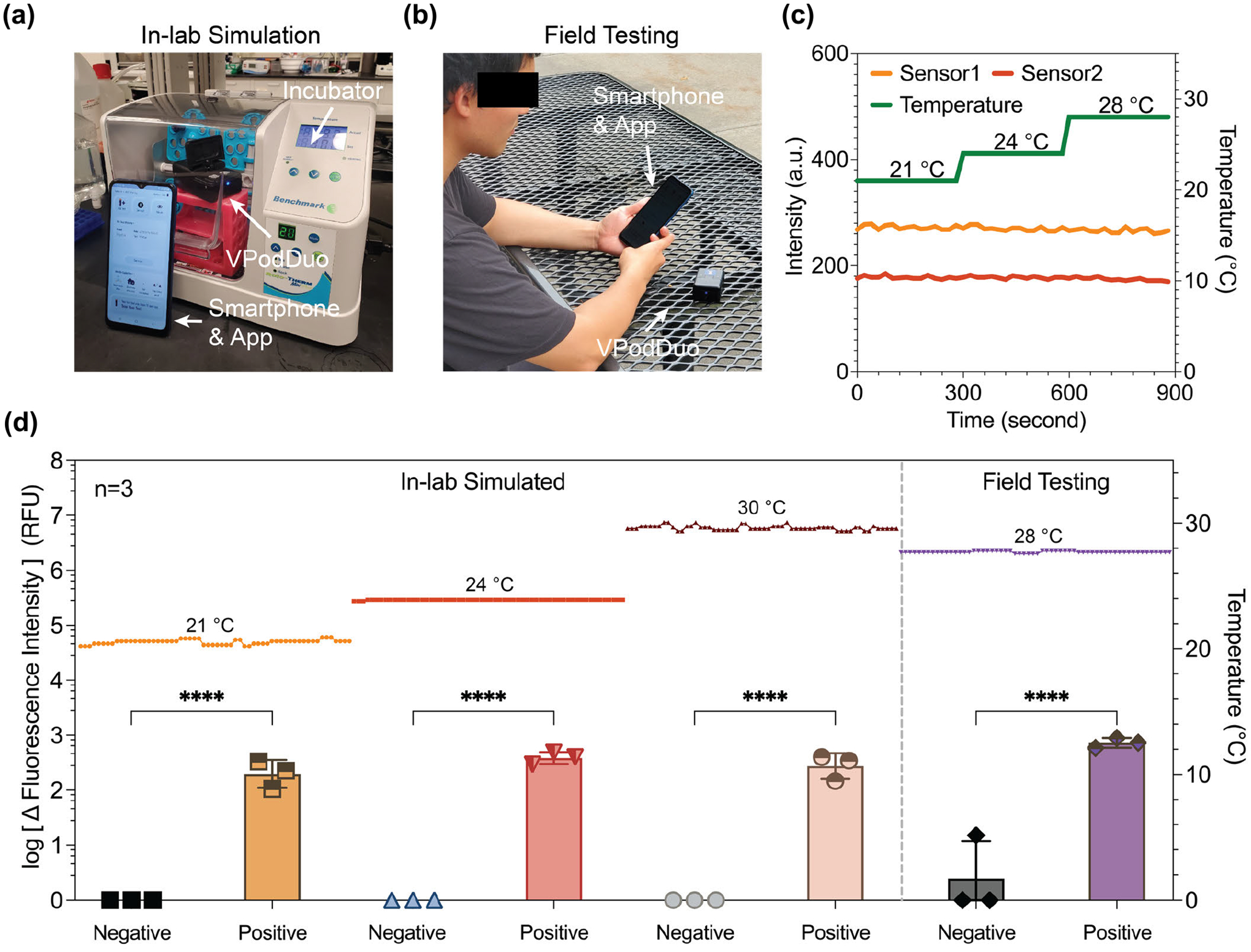
VPodDuo system field-deployability testing using TRAP assay in detecting miR-375–3p. (a) and (b) Show the images of the experimental setups for temperature varying conditions, including in-laboratory simulated and outdoor field conditions, respectively. (c) Laser intensity characterization at ambient temperatures of 21 °C, 24 °C, and 28 °C, demonstrating minimal fluctuation and ensuring reliable fluorescence measurements. (d) TRAP assay detection of 5 nM miR-375–3p in buffer at varying temperatures (21 °C, 24 °C, and 30 °C in-lab, and ~28 °C outdoors). In all conditions, the VPodDuo system successfully distinguished positive samples from negative CTRLs with statistically significant signal differences (****p < 0.0001), confirming robust performance across environments.

To demonstrate the field-deployability of the VPodDuo system in POC settings, we performed the detection of miR-375–3p under varying environmental temperatures, including both laboratory-simulated and outdoor field conditions. For the laboratory simulation, the VPodDuo was placed inside a temperature-controlled incubator set to 21 °C, 24 °C, and 30 °C [Fig. 7(a)], corresponding to cool, comfortably warm, and slightly hot conditions, respectively. Test and CTRL samples were prepared at room temperature (~22 °C) and immediately inserted into the respective ports of the VPodDuo, followed by a 10 min fluorescence measurement. For outdoor testing, the VPodDuo was placed on a bench in an open-air environment [Fig. 7(b)], and pre-prepared samples were similarly measured for 10 min. The ambient outdoor temperature was measured to be approximately 28 °C. As a representative example, the TRAP assay was employed to detect miR-375–3p at a concentration of 5 nM with a final reaction volume of 100 *μ*L.

Fig. 7(c) presents the laser intensity characterization at the tested temperature (21 °C, 24 °C, and 28 °C) in the absence of tubes, confirming minimal fluctuation and ensuring reliable fluorescence detection. The results [Fig. 7(d)] demonstrate that the TRAP assay consistently distinguished positive samples from negative CTRLs across all temperature conditions, with statistically significant signal difference (**** p < 0.0001). These findings validate both the robustness of the TRAP assay and the suitability of the VPodDuo system for molecular cancer diagnostics in diverse POC environments.

## IV. Discussion

We demonstrated the POC molecular detection of various disease-related nucleic acid molecular biomarkers for viruses, bacteria, and cancer, using multiple molecular assays, such as RT-LAMP, RPA, CRISPR/Cas13a, CRISPR/Cas12a, and TRAP. In each case, we intentionally limited the sample incubation time (which generates fluorescence) to only 10 min, to be consistent with the time scale most desired for rapid sample-to-answer POC test requirements. The VPodDuo system exhibited comparable analytical performance to laboratory-based fluorimeters in terms of LOD, validating its potential for portable and low-cost diagnostics. Each of these assays, with the exception of the room-temperature TRAP assay, required a separate heat incubation step before fluorescence measurement due to the absence of an integrated heating block. Rather than adding thermal hardware and CTRL loops that increase device complexity, size, power consumption, and cost, we initially prioritized a streamlined reader architecture that reduces infrastructure burden and supports decentralized deployment, consistent with the affordable, sensitive, specific, user-friendly, rapid, robust, equipment-free, deliverable (ASSURED) criteria set by the WHO. While many widely adopted nucleic acid assays (e.g., RT-LAMP, CRISPR/Cas) currently require thermal incubation, our platform was designed to first establish proof-of-concept analytical performance using assays both with and without heat requirements. Looking forward, we envision two complementary strategies to expand VPodDuo’s versatility: 1) further development and optimization of room-temperature amplification assays, such as TRAP and 2) integration of a modular, low-power heating unit to support assays requiring heat-incubation for their execution and optimal performance.

Beyond the current lack of integrated heat-incubation functionality, the VPodDuo still has room for improvement. Because it is a closed-loop system within a compact enclosure, heat generated by the electronic circuitry and the laser diode must be carefully managed. Excess heating may reduce the laser diode bandgap energy, causing a slight but measurable shift in the excitation wavelength. Such a shift could increase the detected signal by allowing more excitation light to pass through the optical filters, potentially introducing bias in quantitative measurements. Moreover, although we envision that manufactured devices will be delivered pre-calibrated, user-assisted recalibration may still be required in cases of electrical drift, mechanical misalignment, or other system faults. To address this, we plan to incorporate an LED-driven calibration peripheral that can connect to the VPodDuo via Bluetooth to enable guided, app-based automatic calibration. The customized app will provide a dedicated calibration workflow, and standardized FAM reference solutions at defined concentrations may be offered to verify calibration performance. Finally, the device should be systematically evaluated under high-humidity and elevated-temperature conditions (above 30 °C), as these environmental factors are highly relevant for real-world deployment in regions where viral infections are prevalent.

It is important to emphasize that the development of the VPodDuo system is not intended to substitute current centralized laboratory-based diagnostics, but rather to demonstrate an approach to broaden the spectrum of detectable diseases at POC settings while enabling more frequent, rapid, and accessible testing for enhanced control and management of communicable and non-communicable diseases, including infectious pathogens and cancer. As highlighted by Larremore et al. [58], the effectiveness of infectious disease containment is primarily driven by the frequency of testing and the speed of reporting, rather than by the marginal gains achieved through ultrahigh analytical sensitivity, underscoring the critical role of rapid POC testing. This perspective is further supported by studies indicating that a two-step testing strategy—initial screening with a less accurate POC testing followed by confirmatory testing with high-analytical-performance laboratory methods—can be both clinically effective and economically advantageous [59], [60], [61].

In this context, VPodDuo is capable of detecting clinically relevant concentrations of pathogens, including ZIKV and MSSA, within reported ranges of 10^7^–10^8^ CFU/mL [62] (approximately 10^9^ copies/mL) and 40–800 000 copies/mL [63], respectively. This indicates its potential utility in clinical and resource-limited environments. Furthermore, we note that the current sensitivity of the system is influenced not only by hardware limitations but also by the efficiency of signal generation from the diagnostic assays themselves, underscoring the importance of ongoing assay development and optimization (Supplementary Fig. 15). In particular, advances in room-temperature signal amplification strategies and sample enrichment techniques hold considerable promise for further improving the system’s sensitivity without compromising its compact, battery-powered design.

Despite the fact that this study focuses on demonstrating nucleic acid detection at POC settings using a standardized quantitative fluorescence platform, this should not be interpreted as an isolated effort or a claim that portability alone defines the field’s direction. Rather, it aligns with broader, ongoing advances aimed at making molecular diagnostics more accessible outside centralized laboratories, including equipment-free DNA amplification readout via flocculation and turbidity [64], [65], [66], [67]. Together, these efforts underscore the growing momentum toward practical, field-deployable nucleic acid testing workflows that complement conventional laboratory diagnostics.

It is also important to note that continuous advances in molecular assay chemistry remain central to improving POC diagnostics. Portable instruments such as VPodDuo are essential for deployment and accessibility; however, the upper bound of diagnostic performance, especially under short time-to-answer constraints, is largely determined by assay kinetics and signal generation. In this context, ongoing assay development and optimization (e.g., primer/enzyme formulation, temperature and buffer conditions, robustness to complex matrices, suppression of nonspecific amplification, and faster signal accumulation) will continue to push sensitivity and reliability. Instrument design can further support these gains by enabling stable thermal control and preserving quantitative readout across a wide fluorescence range, but the fundamental ceiling is set by the chemistry–kinetics trade space of the underlying assay reactions.

Compared with commercial platforms such as QuantStudio^1^, VPodDuo exhibits a reduced quantitative response in terms of signal change per decade of concentration (i.e., concentration sensitivity). This difference primarily arises from the intrinsic sensitivity and dynamic headroom of the photodiode-based detector used in VPodDuo. In contrast, commercial instruments often employ higher-sensitivity detection architectures (e.g., avalanche photodiodes or photomultiplier tubes) and optimized optics/electronics to maximize photon collection and amplify weak fluorescence signals. These design choices improve sensitivity, but they come with substantial cost and system complexity. VPodDuo instead prioritizes low-cost, readily available components to minimize hardware cost while maintaining comparable analytical performance for target detection, enabling broader accessibility and adoption of molecular assays in POC settings.

The Model List of Essential In Vitro Diagnostics (EDL) (Supplementary Table VIII) from the WHO provides a prioritized catalog of diseases and their recommended in vitro diagnostic (IVD) assays, intended to guide national implementation and strengthen healthcare systems globally. Among the listed diseases, infectious viruses, bacterial infections, and cancers—including HIV, hepatitis B virus (HBV), hepatitis C virus (HCV), hepatitis E virus (HEV), human papillomavirus (HPV), ZIKV, dengue virus, influenza, Mycobacterium tuberculosis, and EGFR mutations—are specifically identified as requiring nucleic acid testing, collectively accounting for approximately 10% of the EDL. Through the development of room-temperature amplification assays, such as TRAP, and the introduction of novel portable diagnostic platforms such as the VPodDuo, we envision addressing this critical subset by enabling decentralized, low-resource-compatible molecular testing.

Supplementary Table IX lists the intra-assay coefficient of variance (CV) for each demonstrated assay, which was performed using buffer. While the current validation focused on buffer matrices under controlled laboratory conditions, we envision extending future studies to include evaluations using real-world biological samples such as blood, saliva, or urine alongside assessments of intra- and interassay reproducibility. However, such investigations will require careful and deliberate development of novel sample preprocessing methods (as exemplified by Liu et al. [68]) as well as thorough assay optimization tailored for POC application to minimize sample-induced variability. Importantly, the sample processing approaches must preserve operational simplicity and robustness to enable use by non-expert personnel without reliance on laboratory equipment. This remains an actively researched area, representing a key frontier for the practical translation of advanced molecular diagnostics to decentralized settings.

To attain broad adoption in modern healthcare, POC molecular testing requires a demanding combination of sensitivity, quantitative accuracy, simplicity, robustness, low cost, and speed. Achieving these goals requires integration of technological advances in multidisciplinary fields that include biochemistry, instrumentation, and data science. As demonstrated in this study, the integration of advanced molecular assays with portable fluorescence detection platforms, such as VPodDuo, represents a significant step toward decentralized and accessible diagnostics, particularly for underserved populations and resource-limited scenarios. The continued efforts in biosensing technology to integrate advanced optics, biochemistry, and artificial intelligence (AI) hold great promise for the realization of fully automated, highly sensitive, and accurate POC molecular diagnostic systems.

## V. Conclusion

In this study, we present the development and fabrication of an inexpensive, portable, pocket-sized fluorimeter with a dual-port configuration, termed VPodDuo, and demonstrate its ability to detect multiple disease-related biomarkers using various molecular assays for POC diagnostics. The integration of an MCU with Bluetooth connectivity enables real-time data transfer to a customized smartphone application, facilitating remote data sharing and monitoring for telemedicine applications, particularly in resource-limited settings. Unlike single-port fluorimeters, which require separate testing of CTRL and test samples, effectively doubling the testing time, the VPodDuo system enables simultaneous measurement of both samples, eliminating the need for separate CTRL testing and significantly improving efficiency. Despite its compact and portable design, the VPodDuo achieves analytical performance comparable to laboratory-based fluorimeters, which typically require bulky instrumentation, desktop computers, and continuous power from wall outlets. We envision that the VPodDuo system, when integrated with a room temperature assay, such as TRAP, may potentially be applied in real-world healthcare settings to enable rapid, accessible, and cost-effective molecular diagnostics of various diseases, ultimately enhancing patient outcomes and bridging the healthcare gaps across the globe.

## Supplementary Material

supp2-3693175

supp1-3693175

This article has supplementary downloadable material available at https://doi.org/10.1109/JSEN.2026.3693175, provided by the authors.

## Figures and Tables

**TABLE I:** Summary of the Demonstrated Assays Targeting Different Biomarkers and Their Respective Detection Limits Achieved Using Different Commercial Laboratory-Based Fluorimeters and VPodDuo

Target	Type of Assay	Commercial Equipment Used for Comparison	Limit of Detection (Commercial)	Limit of Detection (VPodDuo)
ZIKV RNA	RT-LAMP	QuantStudio^™^	**10**^**4**^ ***copies*/*μL***	**10**^**4**^ ***copies*/*μL***
MSSA DNA	RPA	QuantStudio^™^	**10**^**1**^ ***copies*/*μL***	10^2^ *copies*/*μL*
HIV RNA	CRISPR/Cas13a	Microplate reader	7.74 *pM*	**6.83 *pM***
L858R EGFR DNA	CRISPR/Cas12a	Microplate reader	47 *pM*	**29.2 *pM***
miR-375-3p	TRAP	Qubit^™^	**500 *pM***	**500 *pM***

## Footnotes

1 Trademarked.
