# Supplementary material for: A Dual-Port, Smartphone-Linked, Pocket-Size Fluorimeter for Rapid Molecular Diagnostic Assays at Point of Care
Source: IEEE Sens J. Author manuscript; Available in PMC 2026 Aug 6. (PMC13441225; doi:10.1109/jsen.2026.3693175)
Supplement: Supplementary file 1 [file NIHMS2196618-supplement-supp2-3693175.pdf]

Supplementary information

A dual-port, smartphone-linked, pocket-size fluorimeter for rapid molecular diagnostic assays at point-of-care

Hankeun Lee [a,d], Jongwon Lim [b,d], Skye Shepherd [b,d], My Thi Tra Nguyen [b,d,k], Lucas Akin [c,d], Amanda Bacon [b,d], Yasmine Sidavi [l], Max Shurgalin [h], Alban Cobi [h], Andriy Nikolayenko [h], Vladimir Fuflyigin [h], Rashid Bashir [a,b,d,f,i,j], Xing Wang [b,c], and Brian T. Cunningham [a,b,c,d,e,f,g]

[a] Department of Electrical and Computer Engineering, University of Illinois at Urbana-Champaign, Urbana, Illinois, USA.
[b] Department of Bioengineering, University of Illinois at Urbana-Champaign, Urbana, Illinois, USA.
[c] Department of Chemistry, University of Illinois at Urbana-Champaign, Urbana, Illinois, USA.
[d] Nick Holonyak Jr. Micro and Nanotechnology Lab, University of Illinois at Urbana-Champaign, Urbana, Illinois, USA.
[e] Center for Genomic Diagnostics, Woese Institute for Genomic Biology, Urbana, IL, USA.
[f] Carl R. Woese Institute for Genomic Biology, University of Illinois at Urbana-Champaign, Urbana, Illinois, USA.
[g] Grainger College of Engineering, University of Illinois at Urbana-Champaign, Urbana, Illinois, USA.
[h] Gener8 LLC, Wilmington, MA, USA.
[i] Department of Biomedical and Translational Science, Carle Illinois College of Medicine, Urbana, Illinois. USA.
[j] Chan Zuckerberg Biohub Chicago, Illinois, USA.
[k] VinUni-Illinois Smart Health Center, VinUniversity, Hanoi 100000, Vietnam.
[l] Department of Molecular and Cellular Biology, University of Illinois at Urbana–Champaign, Urbana, Illinois, USA.

Supplementary Figures

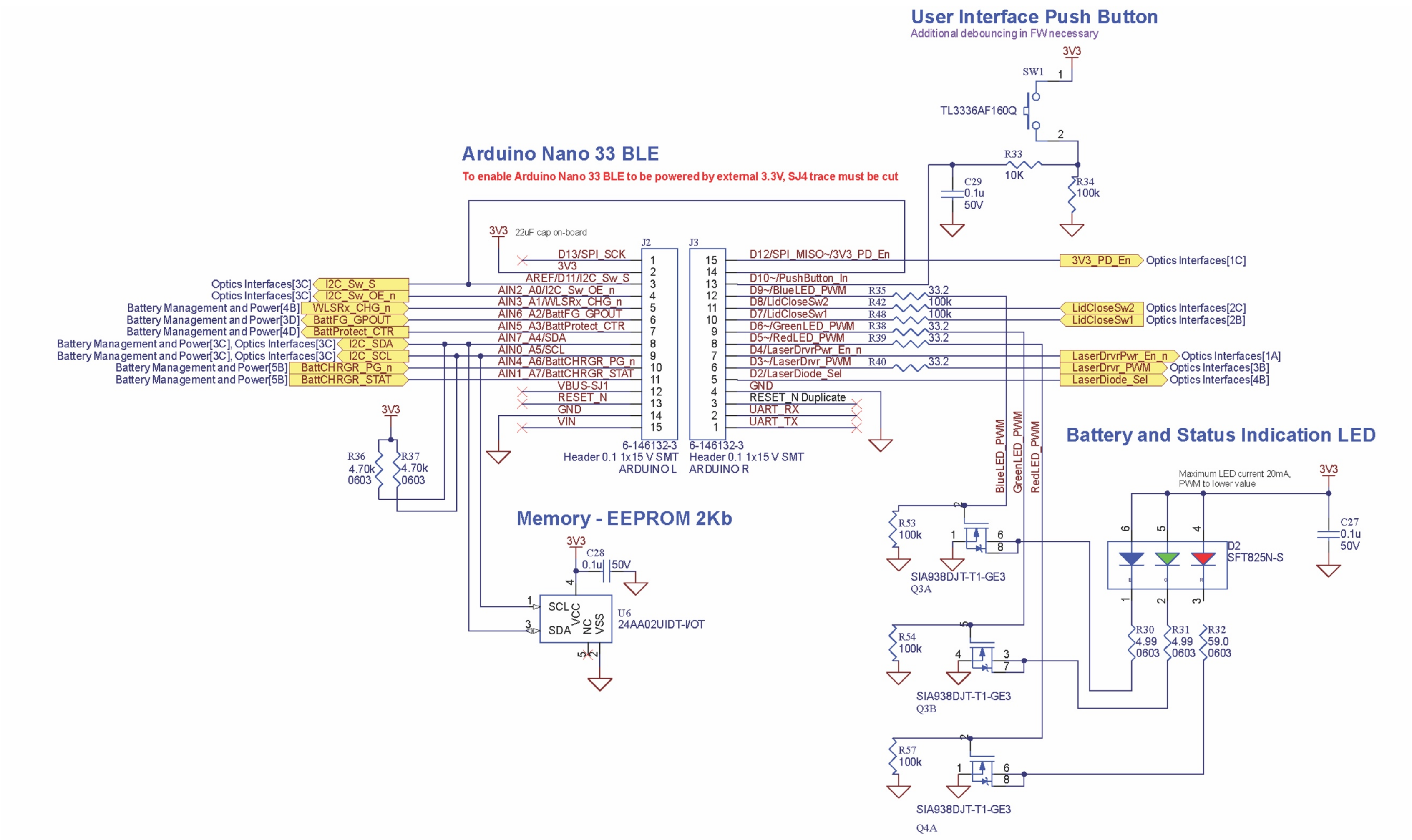

Supplementary Figure 1. Circuit schematic of Arduino Nano 33 BLE and its connections to indicator LED and user interface push button**.**

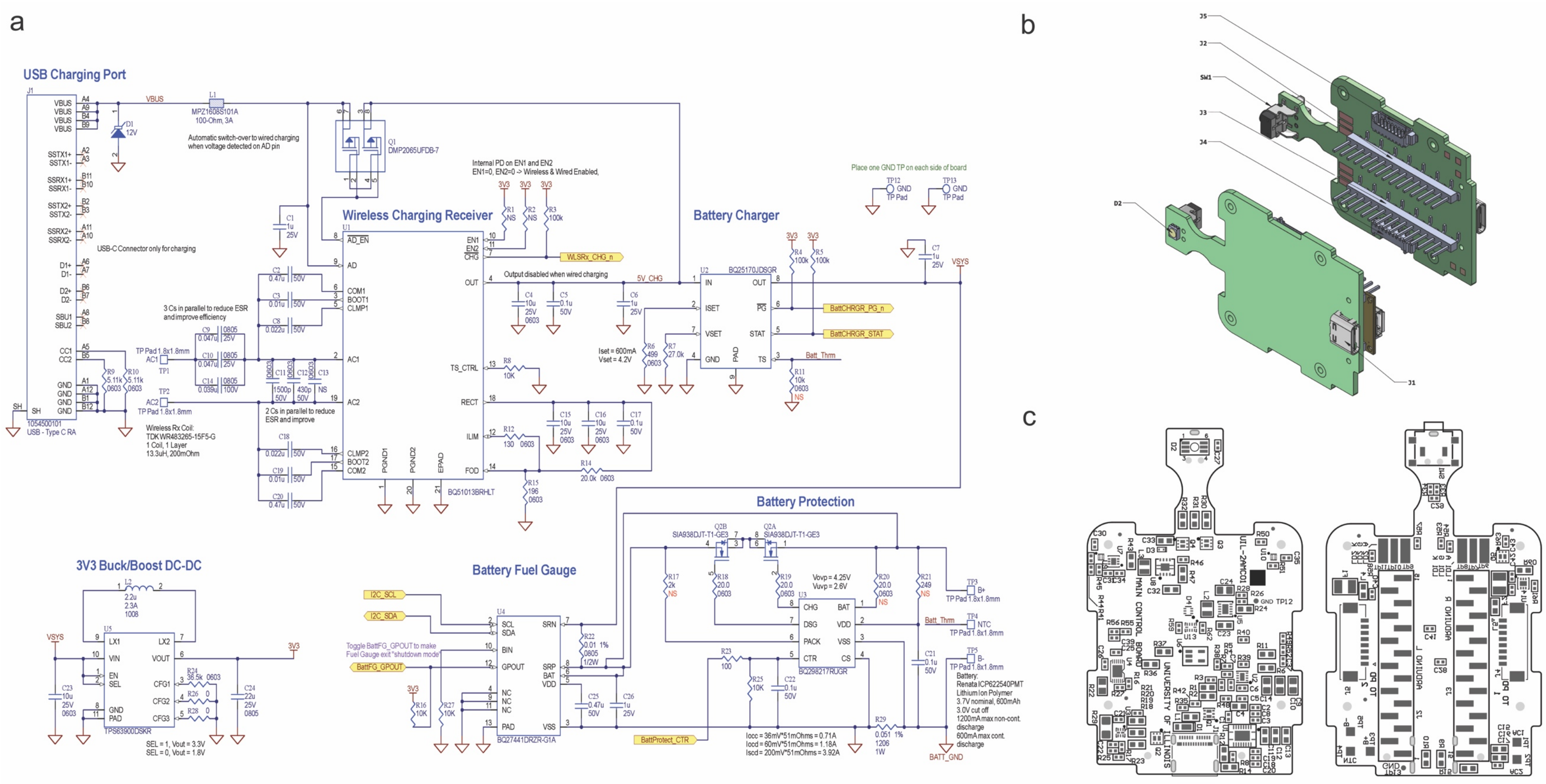

Supplementary Figure 2. Circuit schematic of main PCBA (**a**), rendering of the main PCBA (**b**) and main PCBA layout (**c**).

a

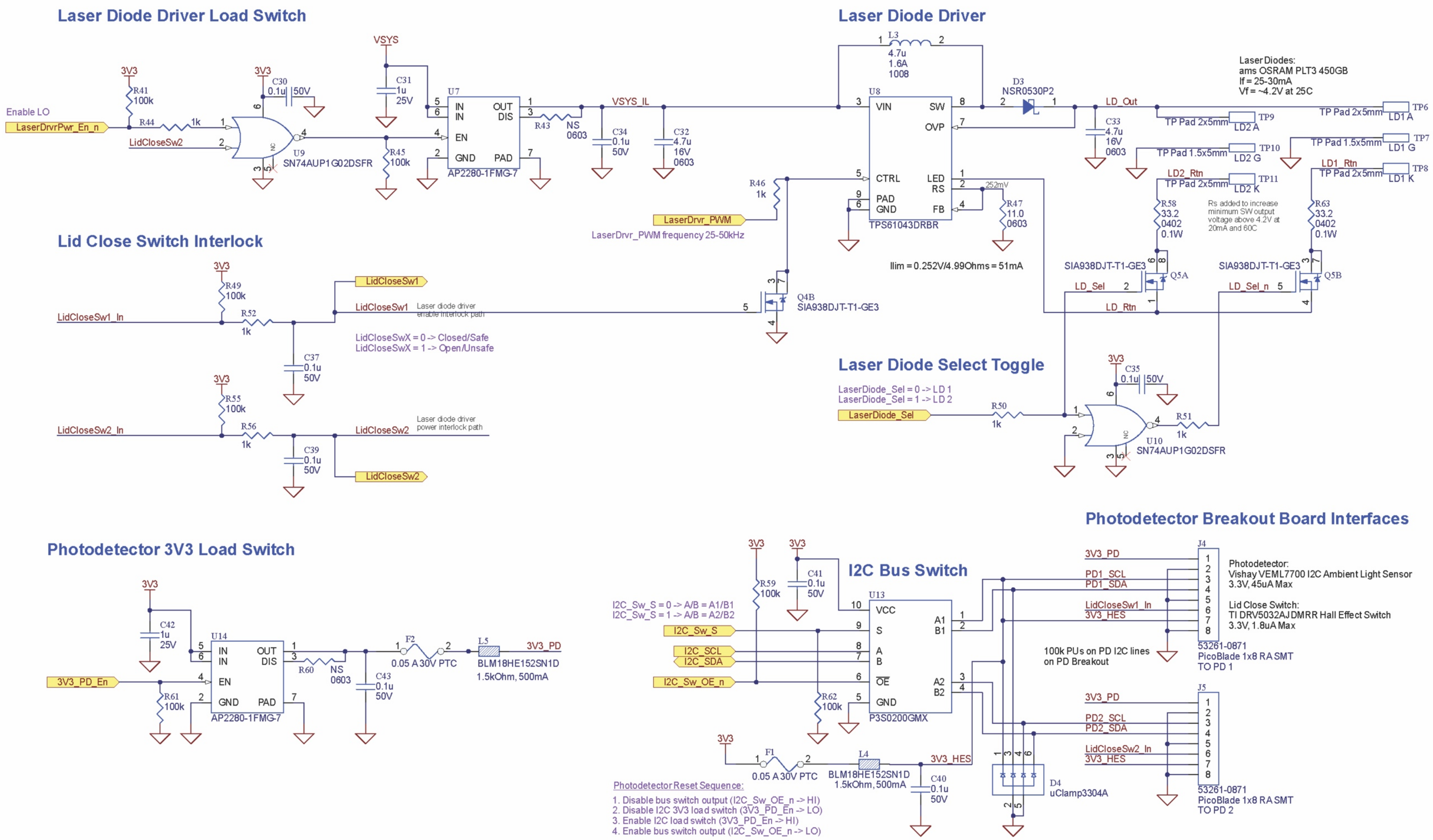

Supplementary Figure 3. Ancillary PCBA of VPodDuo. Circuit schematic of laser diode driver, laser diode load switch, laser diode select toggle, and lid close switch interlock.

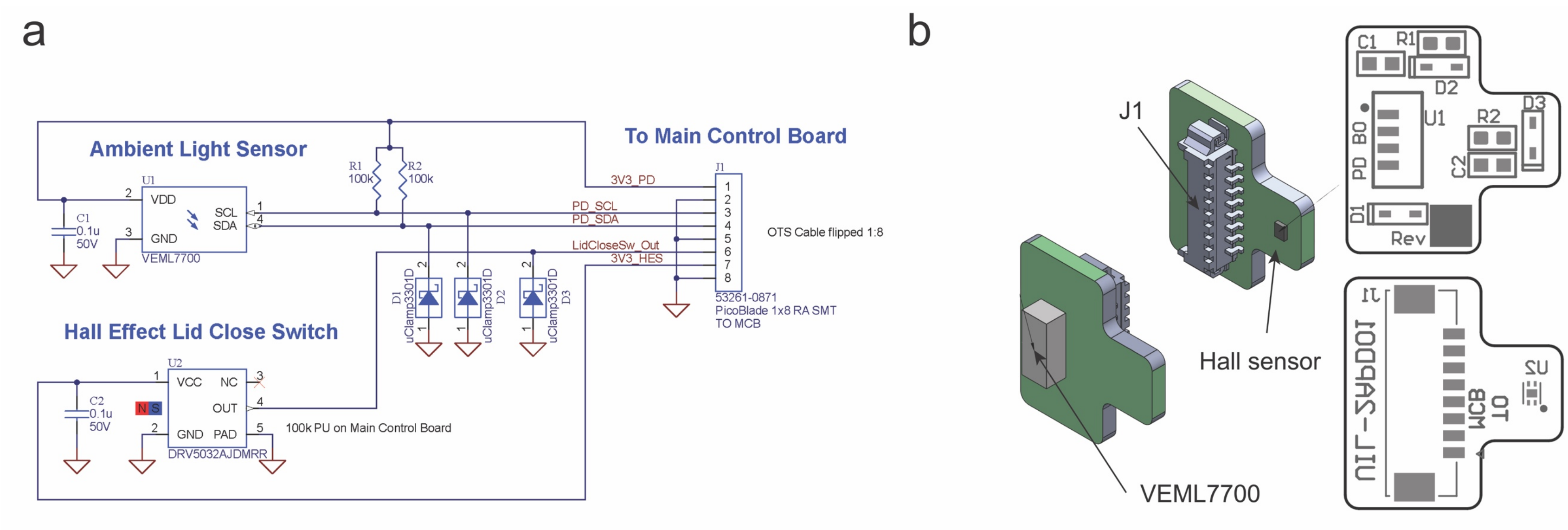

Supplementary Figure 4. a Circuit schematic of ambient light sensor (VEML7700) and hall effect close switch. **b** Rendering of ancillary PCBA and its layout.

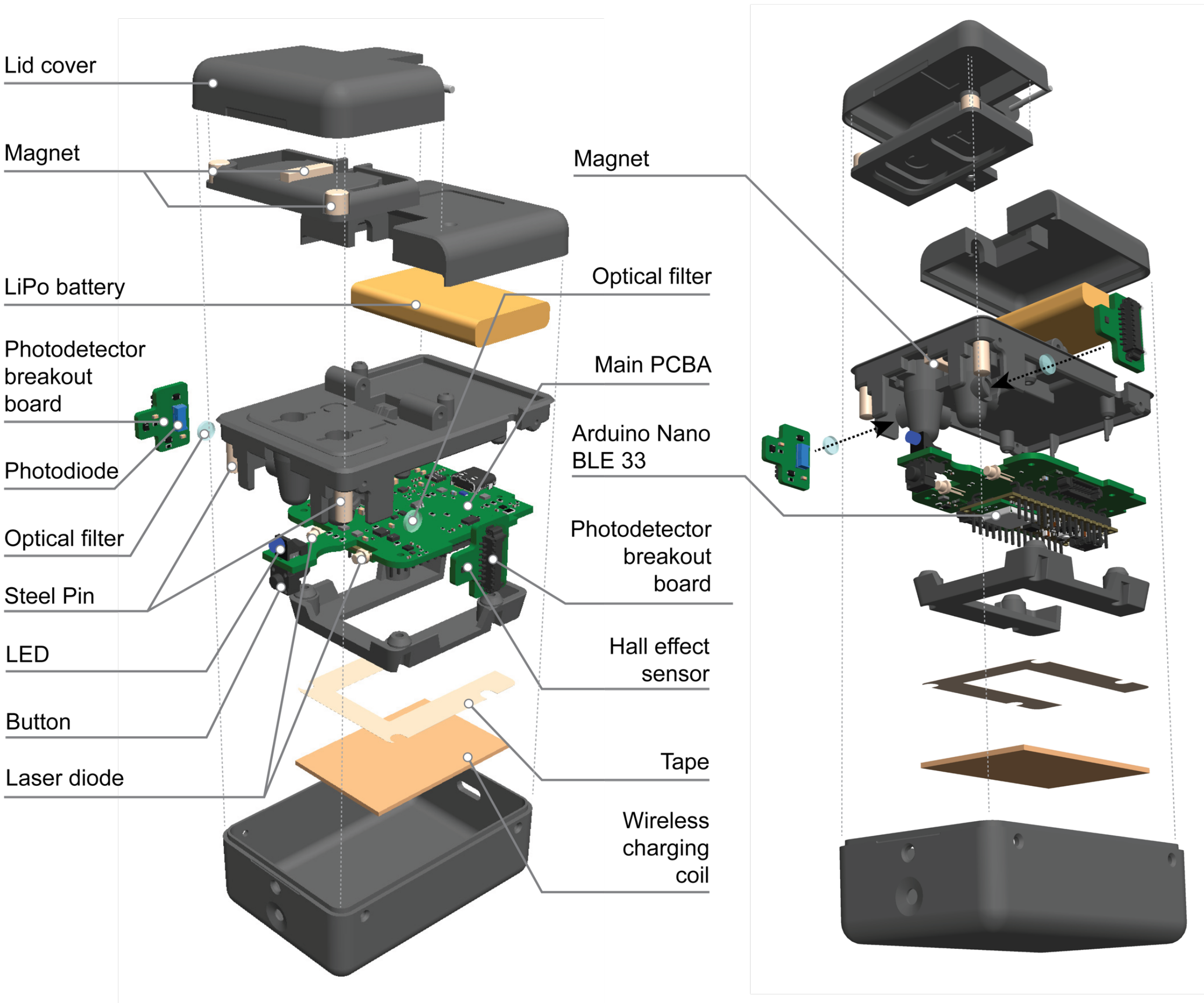

Supplementary Figure 5. Exploded rendering of VPodDuo.

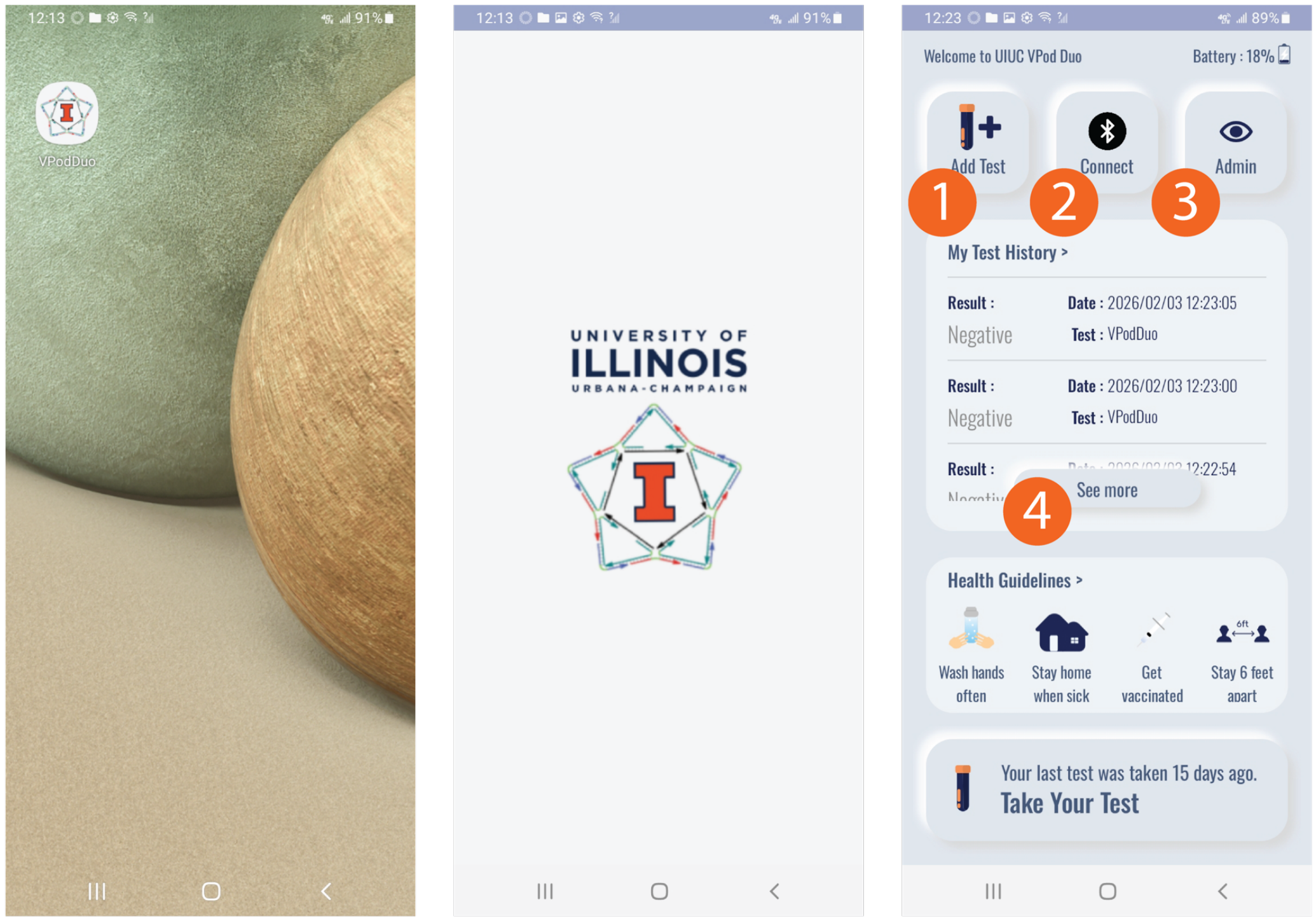

VPodDuo App   Splash Screen   VPodDuo Home Screen

Supplementary Figure 6. Customized smartphone application developed for the VPodDuo system. Detailed views of each numbered screen are provided in **Supplementary Figure 7**.

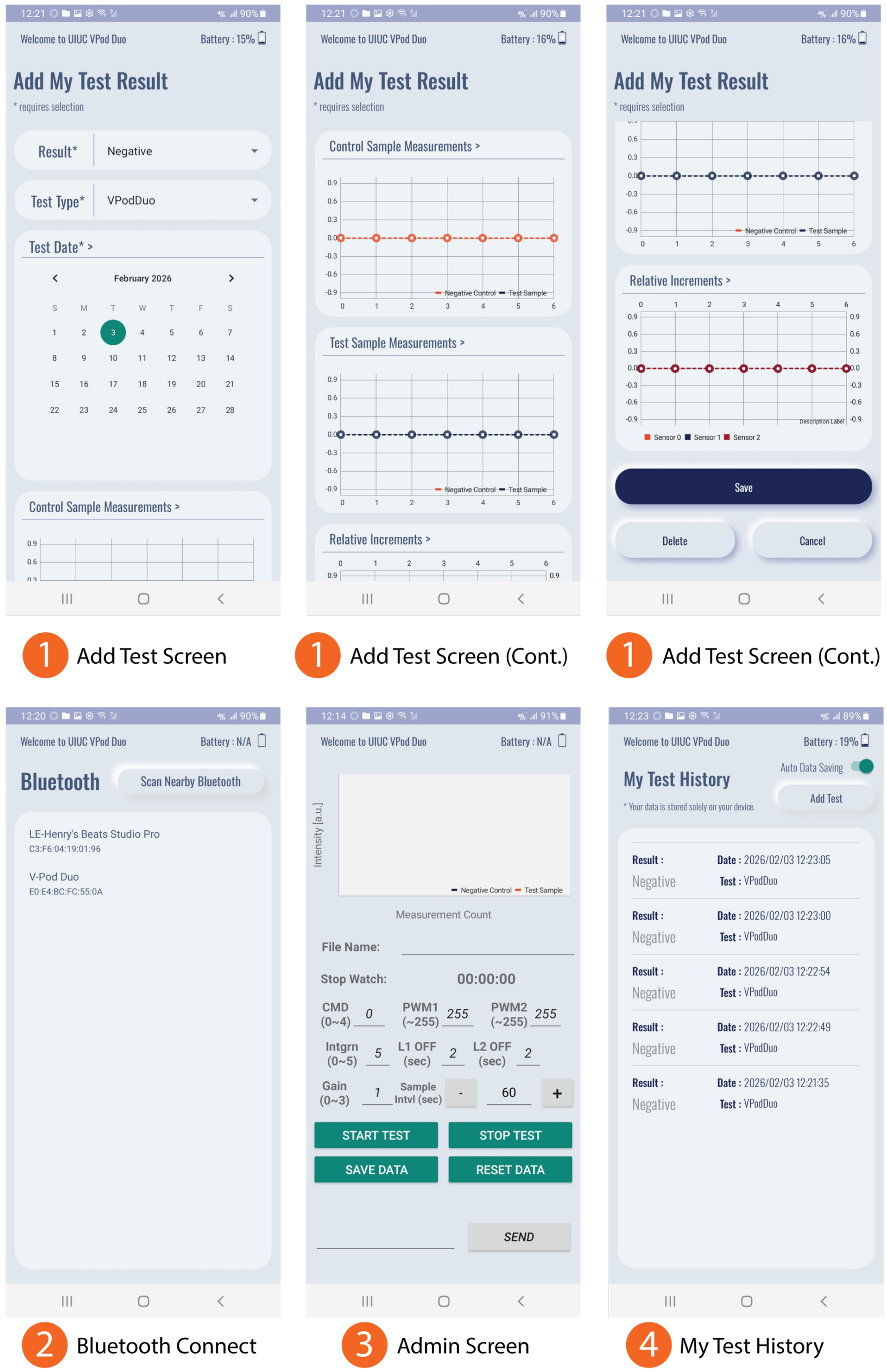

Supplementary Figure 7. Detailed user-interface screens corresponding to Supplementary Figure 6. The "Add Test" screen allows manual entry of test results. The "Bluetooth Connect" screen enables connection to nearby VPodDuo devices. The "Admin" screen is intended for experimental data-collection workflows. Selecting "See more" in the "My Test History" section

opens the full “My Test History” screen. Selecting an individual test then opens the corresponding “Add Test” screen, where the associated raw data are displayed.

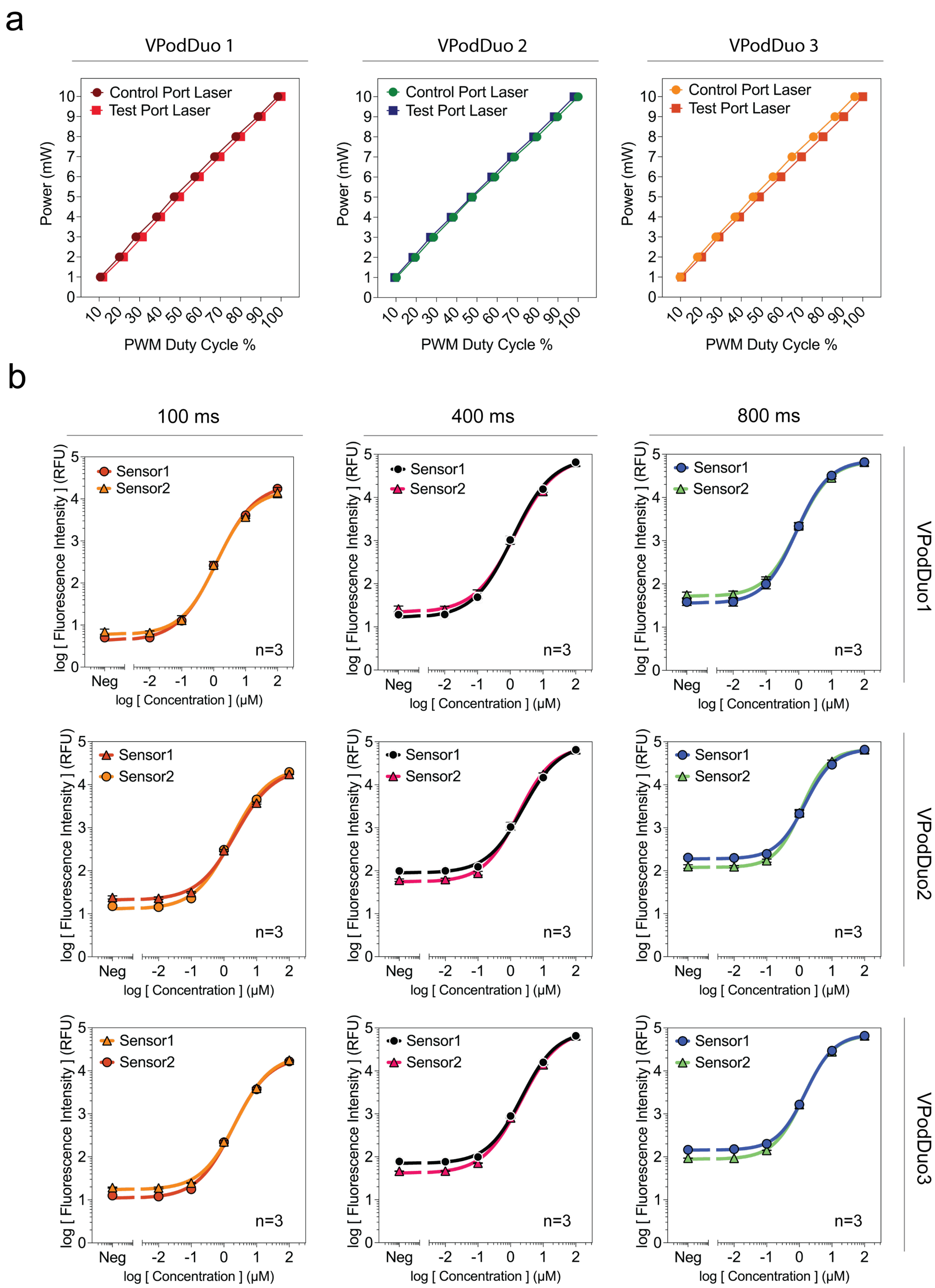

Supplementary Figure 8. VPodDuo laser power modulation and analytical performance evaluation of three different VPodDuo instruments. a Excitation laser power output controlled by pulse width modulation (PWM) duty cycle %. **b** Fluorescence outputs of serially diluted FAM reporters at concentrations 10^2, 10^1, 10^0, 10^{-1}, 10^{-2}, and 0 μM are measured with various sensor integration time settings for three VPodDuo instruments. The columns represent different integration times, while rows represent three different VPodDuo instruments.

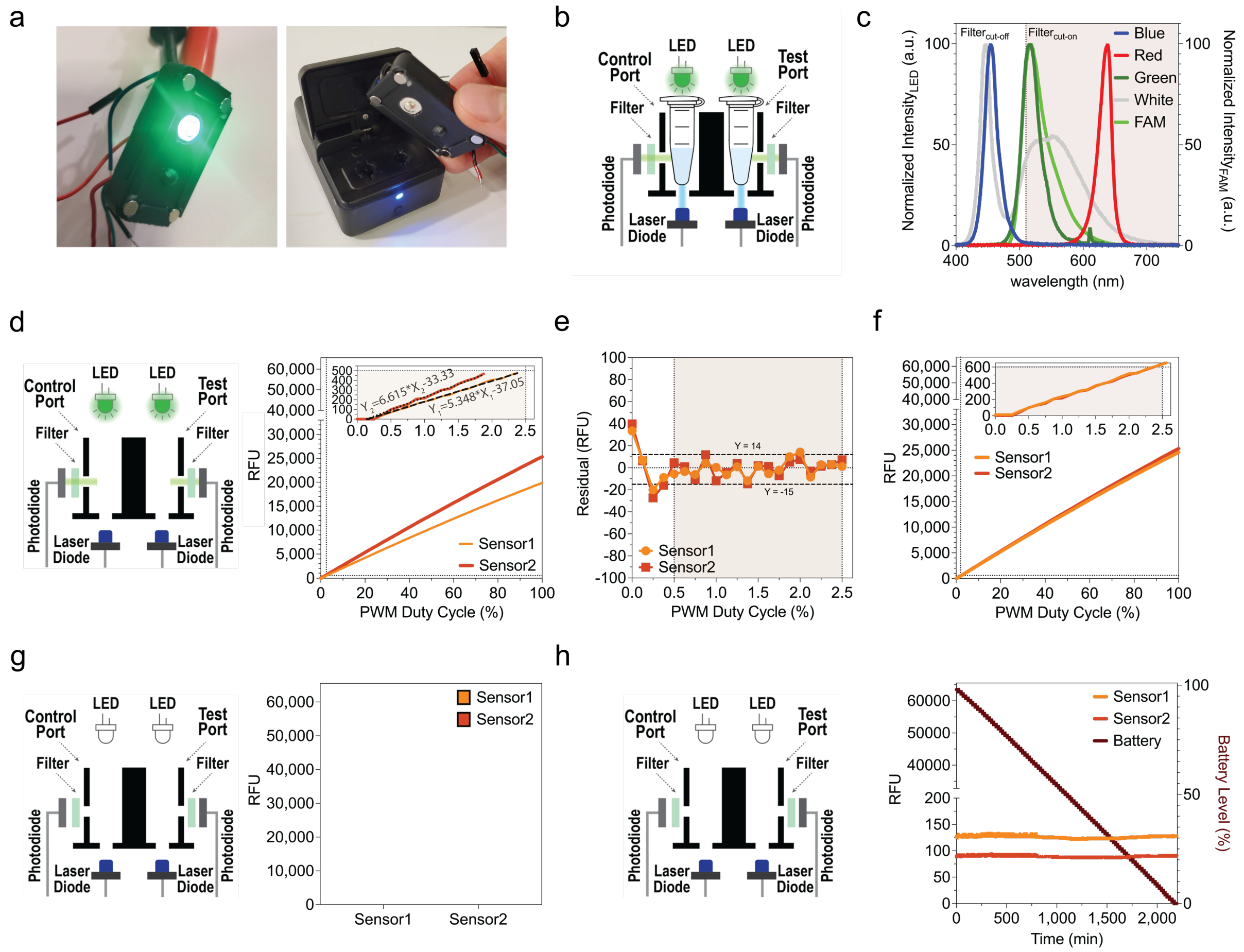

Supplementary Figure 9. VPodDuo dual-port calibration and characterization. a Photograph of the calibration module with high power RGBW LED. **b** Schematic illustration of the calibration working principle. **c** Spectra of red, green, blue, and white light emitted by the high-power LED used for calibration. The green light spectrum closely aligns with the emission spectrum of the FAM fluorophore, which is why its output was selected for photodiode response calibration. **d** Schematic illustration of calibration principle and intensity response of VPodDuo to the green LED across various PWM duty cycle % without test tubes inserted. Sensor1 corresponds to the photodiode from the control port, while Sensor2 corresponds to the photodiode from the test port. Subplot shows the intensity response from 0 – 2.5 PWM duty cycle % with fitted linear lines for each sensor. **e** Residual plot for fitted linear line for both sensors from PWM duty cycle % from 0 to 2.5%. **f** Intensity response after calibration. **g** Schematic illustration of dark current measurement and experimental result. **h** Schematic illustration of VPodDuo longevity characterization and its experimental result.

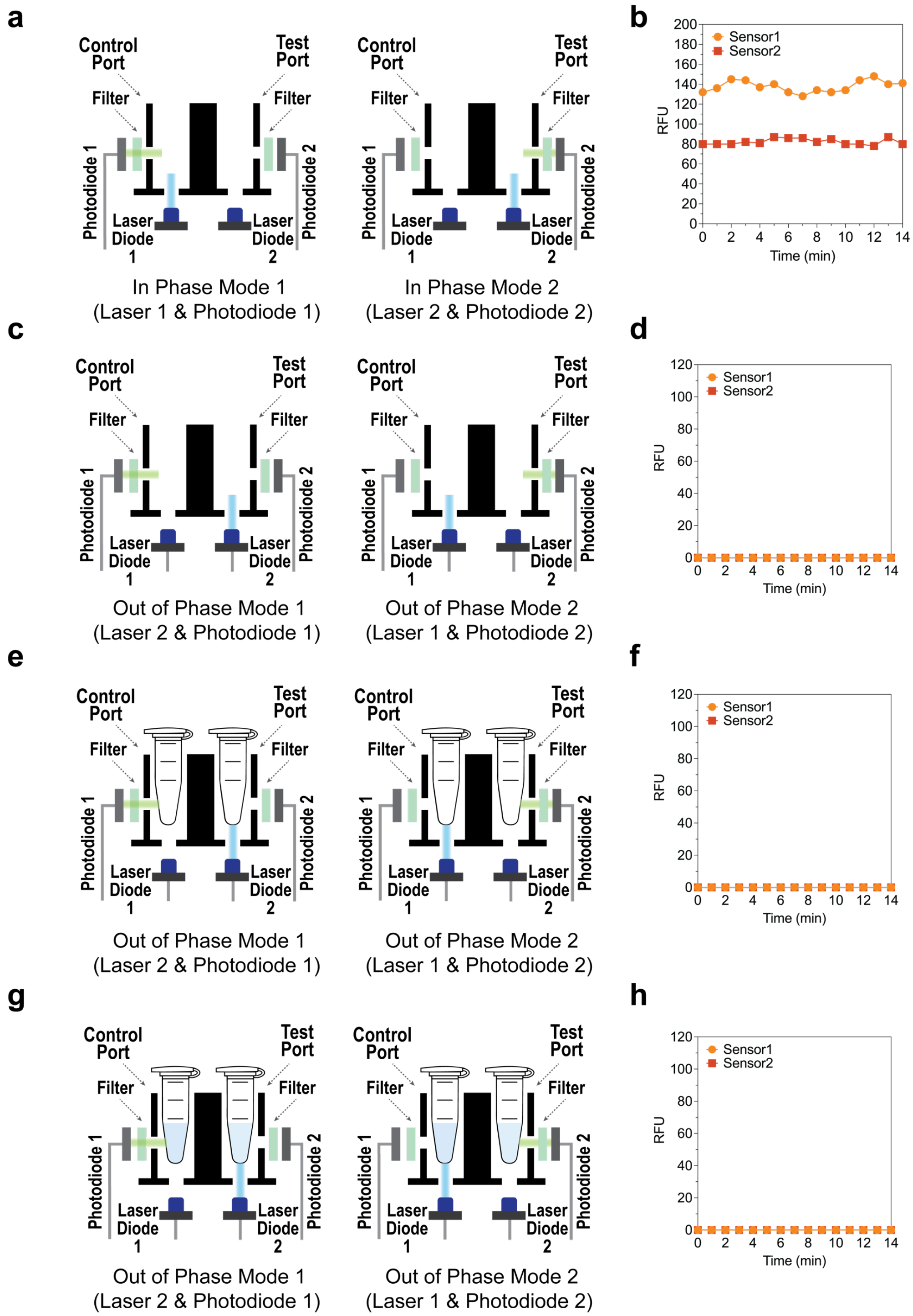

Supplementary Figure 10. Port isolation characterization. The in-phase modes (**a**) correspond to the standard measurement sequence where each port uses its own excitation source and detector (Control Port: Laser 1 with Photodiode 1; Test Port: Laser 2 with Photodiode 2). **b** Representative uncalibrated, in-phase measurements showing non-zero background due to within-port excitation scatter and minor port-ot-port differences in alignment and optical filter transmission. **c, e, g**

Schematic and measurements for the out-of-phase cross-talk test, in which each excitation source is paired with the opposite port's detector (Laser 1 with Photodiode 2; Laser 2 with Photodiode 1) to quantify inter-port optical cross-talk. Out of phase measurements were performed under three conditions – (**c**) empty ports, (**e**) ports with empty tubes, and (**g**) ports with 100 μL DI water. In all cases, the measured signals remained at zero, indicating no detectable inter-port optical cross-talk (**d, f, h**).

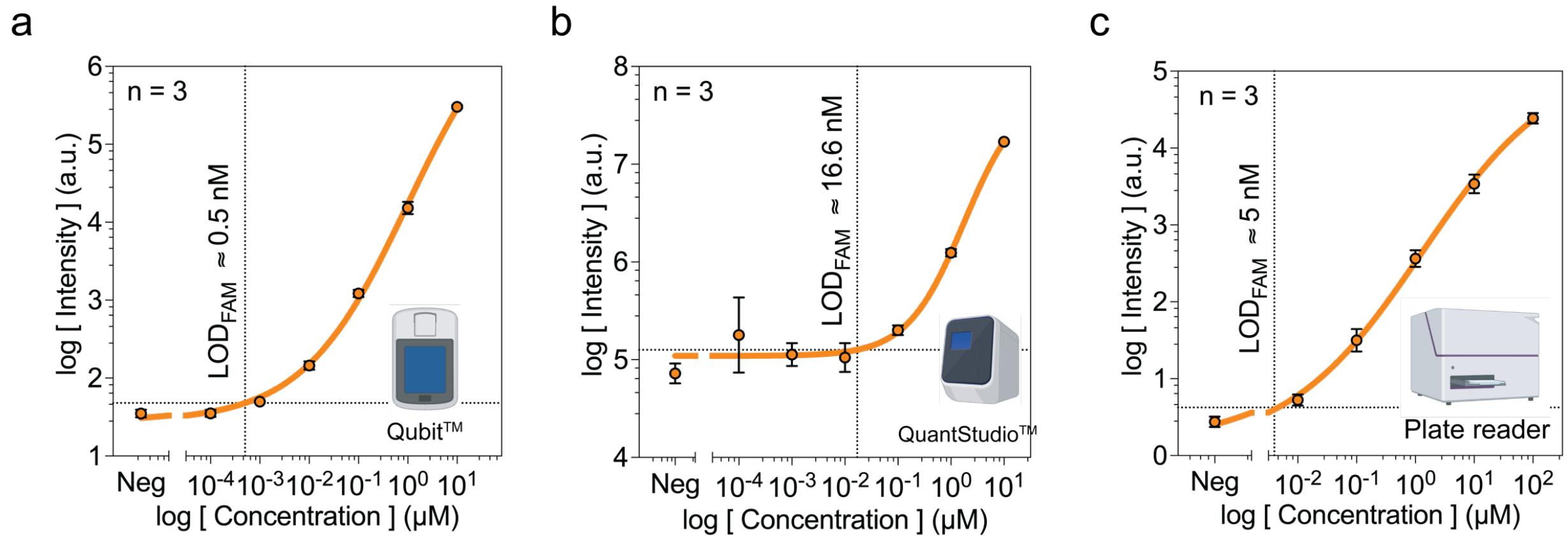

Supplementary Figure 11. FAM concentration dependent responses from three commercially available laboratory fluorimeters including Qubit™ (a), QuantStudio™ (b), and a fluorescence microplate reader (c). Qubit™ exhibited the highest sensitivity while VPodDuo demonstrated comparable analytical performance to QuantStudio™ and the microplate reader.

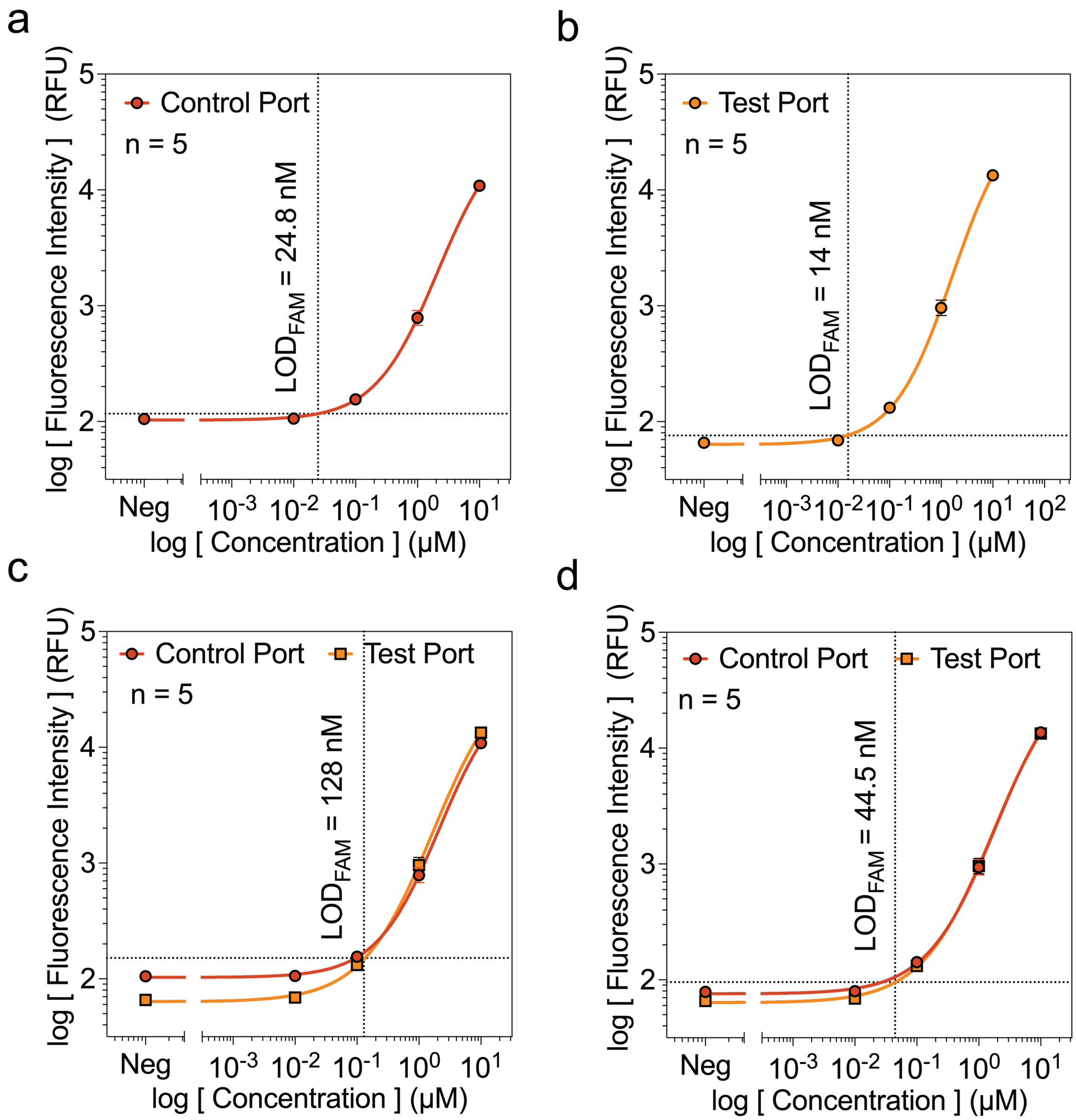

Supplementary Figure 12. Impact of multiple optical sources and calibration on the detection limit in the VPodDuo system. (**a–b**) Independent detection limits for serially diluted FAM were determined to be 24.8 nM and 14 nM for the control and test ports, respectively. (**c–d**) When the negative control measurement from the control port was used as a reference for the test port, the detection limit increased significantly to 128 nM in the absence of calibration. After applying calibration, the detection limit improved to 44.5 nM, highlighting the importance of calibration in maintaining analytical sensitivity in the dual-port VPodDuo configuration.

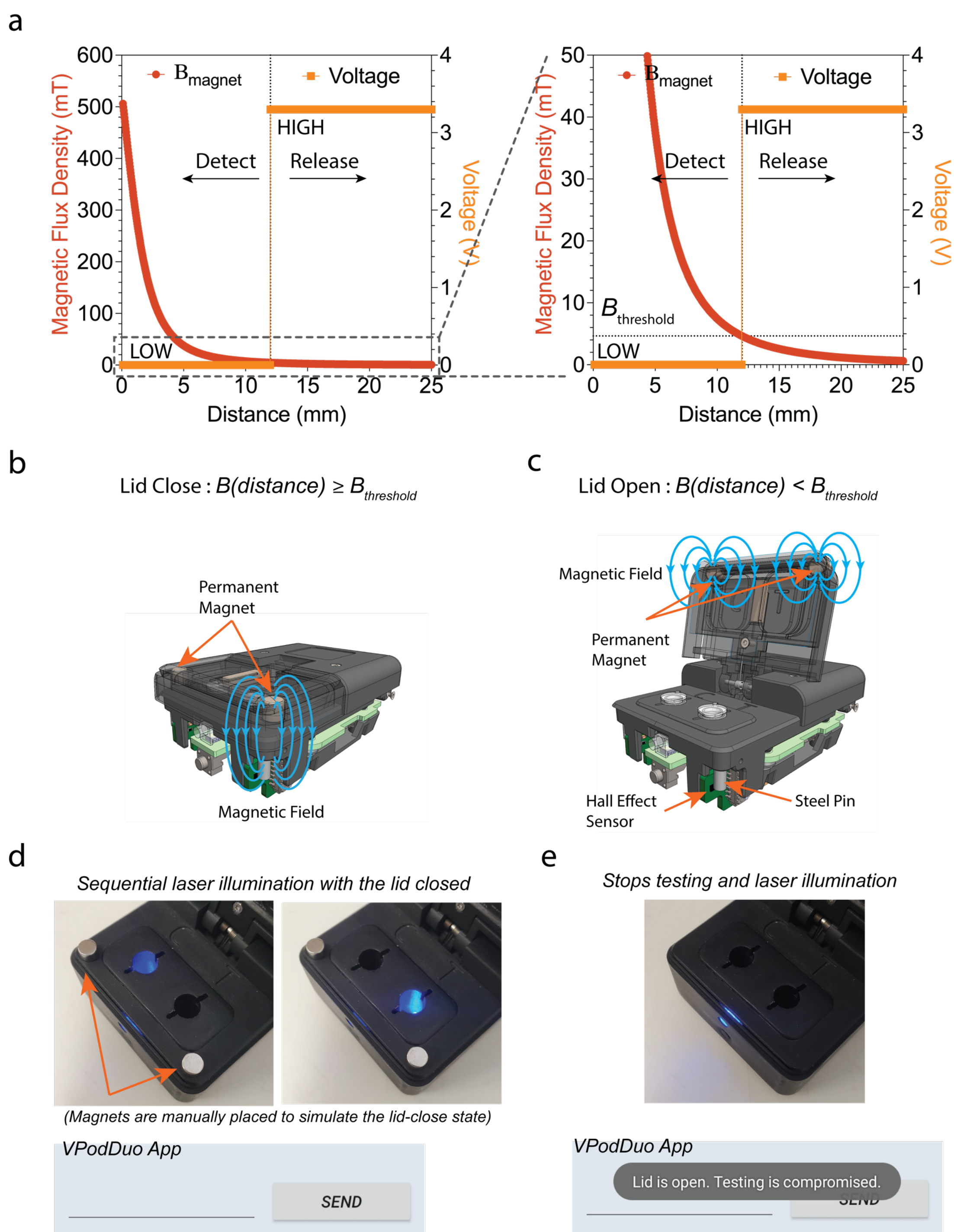

Supplementary Figure 13. Hall-effect lid interlock: characterization and implementation. a Working principle of the Hall-effect switch. The local magnetic flux density at the sensor is a function of magnet to sensor distance. As the magnet approaches the sensor, the local flux density increases. The sensor output is high impedance (released) when the local flux density magnitude is below the operating threshold ($B_{threshold}$). When the local flux density exceeds $B_{threshold}$

(detect), the sensor actively pulls the output low (open-drain). **b-c** Mechanical implementation using a flux concentrator. A steel dowel pin is positioned adjacent to the Hall sensor, while a permanent magnet is embedded in the lid. When the lid is closed, the magnet couples to the steel pin, which concentrates magnetic flux near the sensor and increases the effective field at the sensing element. When the lid is open, the magnet is separated from the pin, and the local flux at the sensor falls below threshold. **d-e** Demonstration of laser interlock operation. Two magnets were manually placed to emulate the lid-closed state. Under the lid-closed condition, the laser illuminates sequentially according to the programmed illumination protocol. When the magnet is removed (lid-open condition), laser illumination stops immediately, and the user is notified via the custom mobile application.

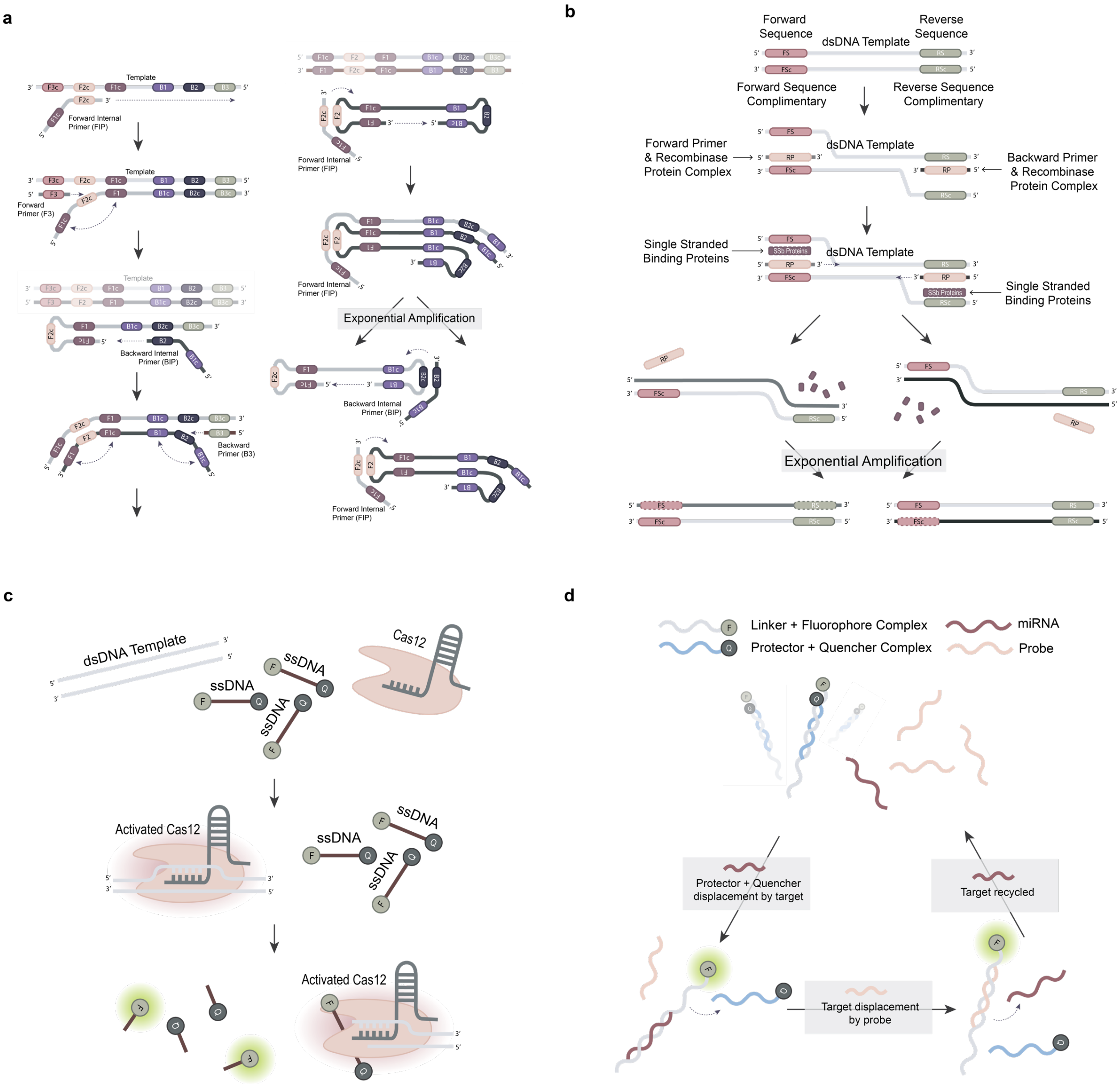

Supplementary Figure 14. Schematic illustration of the working principles of the assays employed in the study. a RT-LAMP. **b** RPA. **c** CRISPR/Cas-based assay. **d** TRAP assay.

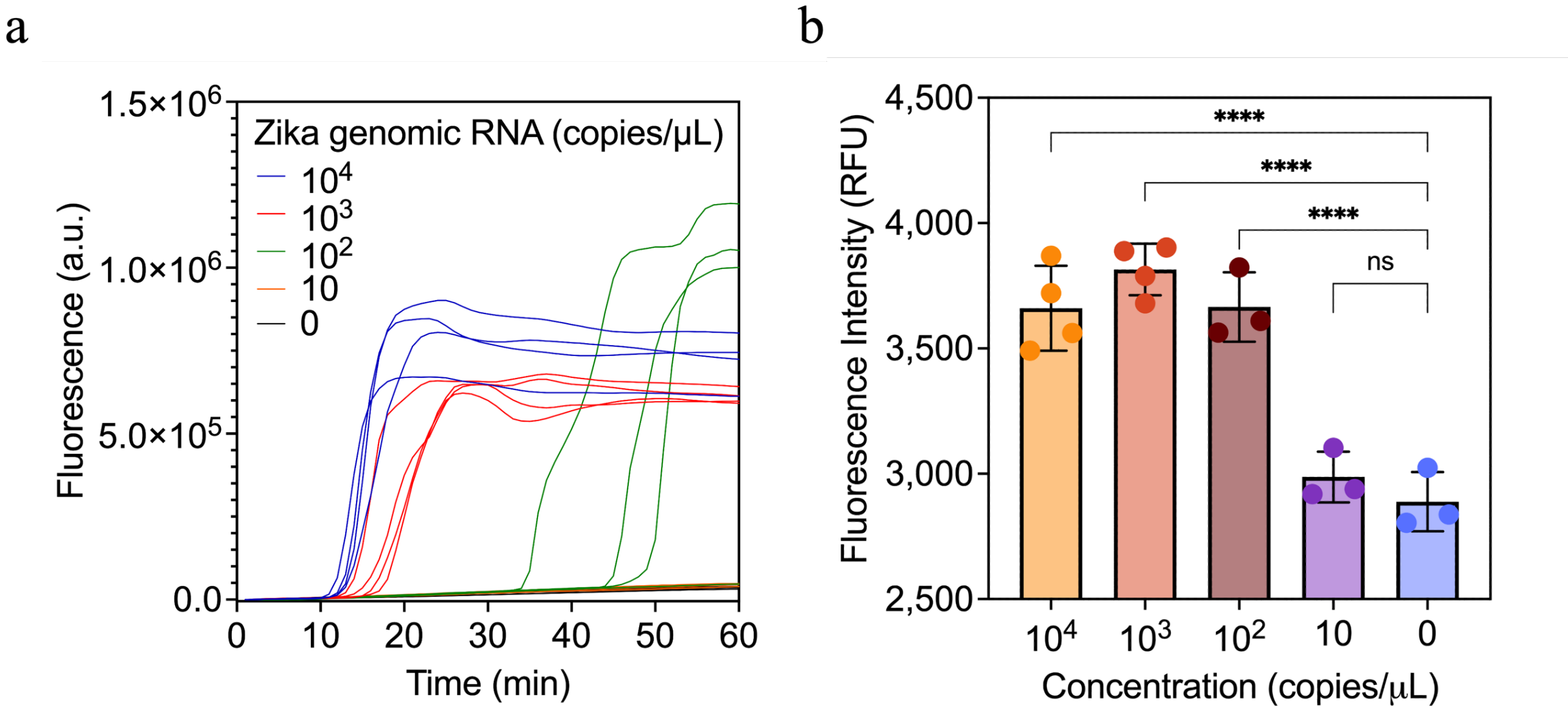

Supplementary Figure 15. Sensitivity of RT-LAMP for ZIKV detection following a full 1-hour incubation. **a** Kinetic fluorescence data collected using the QuantStudio™ system. **b** Endpoint fluorescence data from the same samples collected using the VPodDuo. These results support the conclusion that the observed limit of detection is governed primarily by assay kinetics and incubation conditions, with VPodDuo serving as a portable quantitative readout rather than a direct enhancer of assay-limited sensitivity. ****, $p < 0.0001$.

Supplementary Tables

Index	Quantity	Ref Des	Description	Footprint	Mfg	Mfg PN
1	1		PCB, Main Control Board			
2	2	C3, C19	CAP CER 0.01uF 50V X7R	0402	Murata	GRM155R71H103KA88D
3	15	C5, C17, C21, C22, C27, C28, C29, C30, C34, C35, C37, C39, C40, C41, C43	CAP CER 0.1uF 50V X7R	0402	TDK Corporation	C1005X7R1H104K050BB
4	6	C1, C6, C7, C26, C31, C42	CAP CER 1.0 UF 25V X5R 0402	0402	Taiyo Yuden	TMK105BJ105MV-F
					TDK Corporation	C1005X5R1E105M050BC
					TDK Corporation	CGB2A1X5R1E105K033BC
5	2	C8, C18	CAP CER 0.022UF 50V X7R 0402	0402	Murata Electronics	GCM155R71H223KA55D
6	3	C2, C20, C25	CAP CER 0.47UF 50V X5R 0402	0402	Murata Electronics	GRM155R61H474KE11D
7	1	C13	NO STUFF	0603		
8	4	C4, C15, C16, C23	CAP CER 10 UF 25V X5R 0603	0603	Murata Electronics North America	GRM188R61E106MA73D
					TDK Corporation	C1608X5R1E106M080AC
9	1	C11	CAP CER 1500pF 50V NP0 5%	0603	Murata	GRM1885C1H152JA01D
10	1	C12	CAP CER 430pF 50V NP0 5%	0603	Murata	GRM1885C1H431JA01D
11	2	C32, C33	CAP CER 4.7UF 16V X5R	0603	Murata	GRM188R61C475KE11D
12	1	C24	CAP CER 22UF 25V X5R 0805	0805	Samsung Electro-Mechanics America, Inc.	CL21A226MAQNNNE
13	1	C14	CAP CER 0.039UF 100V C0G 0805	0805	Murata Electronics	GRM2195C2A393JE01J
14	2	C9, C10	CAP CER 0.047uF 25V NP0	0805	Kemet	C0805C473J3GACTU
15	1	D3	DIODE SCHOTTKY 30V 0.5A		ON Semiconductor	NSR0530P2T5G
16	1	D1	TVS UNI 12V	SOD323	Toshiba Semiconductor and Storage	CUHZ12V,H3F
17	1	D2	LED RGB DIFF 3528 6PLCC	PLCC	Seoul Semiconductor Inc.	SFT825N-S
18	1	D4	ESD PROTECTION 3.3V LOGIC QUAD		Semtech Corporation	uClamp3304A
19	2	J2, J3	CONN HEADER VERT 15POS 2.54MM	Other	TE Connectivity AMP Connectors	6-146132-3
20	1	J1	CONN RCPT USB C 3.1 SMT R/A	Other	Molex	1054500101
21	2	J4, J5	CONN HEADER 8POS 1.25MM R/A SMD		Molex	532610871
22	2	L4, L5	FERRITE BEAD 1.5K OHM 0603 1LN	0603	Murata Electronics	BLM18HE152SN1D
23	1	L1	FERRITE 100-OHM 3A603	0603	TDK Corporation	MPZ1608S101A
24	1	L2	INDUCTOR 2.2UH 2.3A 1008	1008	Abracon LLC	AOTA-B252010S2R2MT
25	1	L3	INDUCTOR 4.7UH 1.6A 1008	1008	Abracon LLC	AOTA-B252010S4R7MT
26	4	Q2, Q3, Q4, Q5	NMOS DUAL 20V 4.5A SC70-6	SC70 (SC89, SOT	Vishay Siliconix	SIA938DJT-T1-GE3
27	1	Q1	PMOS DUAL 20V 4.5A 6-UDFN	DFN (TDFN, UDFN	Diodes Incorporated	DMP2065UFDB-7
28	2	R26, R28	JUMPER 0.000 OHM 0402	0402	Panasonic Electronic Components	ERJ-2GE0R00X
29	2	R17, R21	NO STUFF 0402	0402		
30	1	R23	RES 100 OHM 1% 0402	0402	Panasonic Electronic Components	ERJ-2RKF1000X
31	6	R44, R46, R50, R51, R52, R56	RES 1.00 KOHM 1% 0402	0402	Panasonic Electronic Components	ERJ-2RKF1001X
32	5	R8, R16, R25, R27, R33	RES 10.0 KOHM 1% 0402	0402	Panasonic Electronic Components	ERJ-2RKF1002X
33	16	R3, R4, R5, R34, R41, R42, R45, R48, R49, R53, R54, R55, R57, R59, R61, R62	RES 100 KOHM 1% 0402	0402	Panasonic Electronic Components	ERJ-2RKF1003X
34	1	R7	RES 27.0 KOHM 1% 0402	0402	Panasonic Electronic Components	ERJ-2RKF2702X
35	6	R35, R38, R39, R40, R58, R63	RES 33.2 OHM 1% 0402	0402	Panasonic Electronic Components	ERJ-2RKF33R2X
36	6	R1, R2, R43, R60, R11, R20	NO STUFF 0603	0603		
37	1	R47	RES 11.0 OHM 1% 0603	0603	Panasonic Electronic Components	ERJ-3EKF11R0V
38	1	R12	RES 130 OHM 1% 0603	0603	Panasonic Electronic Components	ERJ-3EKF1300V
39	1	R15	RES 196 OHM 1% 0603	0603	Panasonic Electronic Components	ERJ-3EKF1960V
40	1	R14	RES 20.0 KOHM 1% 0603	0603	Panasonic Electronic Components	ERJ-3EKF2002V
41	2	R18, R19	RES 20.0 OHM 1% 0603	0603	Panasonic Electronic Components	ERJ-3EKF20R0V
42	1	R24	RES 36.5 KOHM 1% 0603	0603	Panasonic Electronic Components	ERJ-3EKF3652V
43	2	R36, R37	RES 4.70 KOHM 1% 0603	0603	Panasonic Electronic Components	ERJ-3EKF4701V
44	1	R6	RES 499 OHM 1% 0603	0603	Panasonic Electronic Components	ERJ-3EKF4990V
45	2	R30, R31	RES 4.99 OHM 1% 0603	0603	Yageo	RC0603FR-074R99L
46	2	R9, R10	RES 5.11 KOHM 1% 0603	0603	Panasonic Electronic Components	ERJ-3EKF5111V
47	1	R32	RES SMD 59 OHM 1% 1/10W 0603	0603	Panasonic Electronic Components	ERJ-3EKF59R0V
48	1	R22	RES 0.010 OHM 1% 0805	0805	Bourns Inc.	CRF0805-FX-R010ELF
49	1	R29	RES 0.051 OHM 1% 1206 0.5W CURRENT SENSE	1206	Panasonic Electronic Components	ERJ-8BWFR051V
50	2	U7, U14	IC PWR SWITCH P-CH 1:1 6DFN	DFN (TDFN, UDFN	Diodes Incorporated	AP2280-1FMG-7
51	1	U2	IC BATT CHRGR 800mA WSON-8	SON (HSON, TDS	Texas Instruments	BQ25170JDSGR
52	1	U4	IC FUEL GAUGE LI-ION 12SON	SON (HSON, TDS	Texas Instruments	BQ27441DRZR-G1A
53	1	U3	IC BATT PROTECT LI-ION 1-CELL	QFN (UQFN, UFQI	Texas Instruments	BQ298217RUGR
54	1	U1	QI WIRELESS POWER RECEIVER		Texas Instruments	BQ51013BRHLT
55	1	U8	LED DRVR BOOST ADJ 60MA 8-SON	SON (HSON, TDS	Texas Instruments	TPS61043DRBR
56	1	U5	IC REG BCK BST ADJ 400MA 10SON	SON (HSON, TDS	Texas Instruments	TPS63900DSKR
57	1	U6	IC EEPROM 2K I2C 400KHZ SOT23-5	SOT23 (TSOT23)	Microchip Technology	24AA02UIDT-I/OT
58	1	U13	IC I3C I2C SWITCH 2CH XQFN10	QFN (UQFN, UFQI	NXP Semiconductors	P3S0200GMX
59	2	U9, U10	IC GATE NOR 1CH 2-IN 6SON	SON (HSON, TDS	Texas Instruments	SN74AUP1G02DSFR
60	1	SW1	SWITCH TACT SPST-NO RA 0.05A 12V		E-SWITCH	TL3336AF160Q
61	5	TP1, TP2, TP3, TP4, TP5	NO STUFF			
62	4	TP6, TP8, TP9, TP11	NO STUFF			
63	2	TP7, TP10	NO STUFF			
64	2	F1, F2	FUSE PTC RESET 0.05A 30V		Bourns Inc.	MF-USMF005-2

Supplementary Table 1. Bill of material for main PCBA.

Index	Quantity	Ref Des	Description	Footprint	Mfg	Mfg PN
0	1		PCB, Photodetector Breakout			
1	2	C1, C2	CAP CER 0.10 UF 50V X7R 0603	0603	Murata	GRM188R71H104KA93D
					Kemet	C0603C104K5RACTU
					AVX Corporation	06035C104KAT2A
					TDK Corporation	C1608X7R1H104K080AA
					TDK Corporation	CGA3E2X7R1H104K080AE
2	3	D1, D2, D3	ESD PROTECTION 3V LOGIC		Semtech Corporation	uClamp3301D
3	1	J1	CONN HEADER 8POS 1.25MM R/A SMD		Molex Incorporated	532610871
4	2	R1, R2	RES 100 KOHM 1% 0603	0603	Panasonic Electronic Components	ERJ-3EKF1003V
5	1	U2	SWITCH HALL OMNI X2SON	SON (HSON, TDS	Texas Instruments	DRV5032AJDMRR
6	1	U1	SENSOR AMBIENT LIGHT 16-BIT I2C 4-SMD	Other	Vishay Semiconductor Opto Division	VEML7700-TR

Supplementary Table 2. Bill of material for ancillary PCBA.

PRIMER	SEQUENCE
**ZIKA F3**	AATGAGTGACCTGGCTAAGC
**ZIKA B3**	AAAAGACACGAGGCCAAGG
**ZIKA FIP**	CAGCGCCAGATGAGCTACATCTTTTTGATGGGTGCCACCTTC
**ZIKA BIP**	AGCGGCATTCAAAGTCAGACCATTTCACGGGGTGTCCAATT
**ZIKA LF**	CCTCCAGTGTTCATTTCCGC
**ZIKA LB**	GCGTTGCTGGTATCTTTCATCTT

Supplementary Table 3. Primer sequences for RT-LAMP assay

PRIMER	SEQUENCE
**VICK FORWARD PRIMER**	CGTGGACGTATTCGTATCGTCAATGATATGGCACTC
**VICK REVERSE PRIMER**	CACGTGCGATTAGACCTTCTTCTTCATTTAAATC
**VICK PROBE**	AGATGCTTGGTATGGCGAAAGAAGACATCATCGGATAT/iBHQ-1dT/A/idSp/A/iFluorT/GTTAAGTGTATTAAG/3Phos/

Supplementary Table 4. Primers and probe sequences for RPA assay.

NAME	5'-SEQUENCE-3'
**DNA TEMPLATE**	TAA TAC GAC TCA CTA TAG ATA GCA GGA ACT ACT AGT ACC CTT CAG GAA CAA ATA GGA TGG ATG ACA AAT AAT CA CT ATC CCA GTA GGA GAA AT TAT A AGA TG ATA ATC CTG GGA TA AT AAA ATA GTA AGA ATG TAT AGC CCT ACC AGC ATT CTG GAC ATA AGA CAA GGA CCA AAG GAA CCC TTT AGA GAC TAT GTA GAC CGG TTC TAT AAA ACT CTA AGA GCC GAG CAA GCT TCA CAG GAG G
**RNA TEMPLATE**	GAUAGCAGGAACUACUAGUACCCUUCAGGAACAAAUAGG AUGGAUGACAAAUAAUAUCCCAGUAGGAGAAAUUAUAAG AUGAUAAUCCUGGGAUAAUAAAAUAGUAAGAAUGUAUA GCCCUACCAGCAUUCUGGACAUAAGACAAGGACCAAAGG AACCCUUUAGAGACUAUGUAGACCGGUUCUAUAAAACUC UAAGAGCCGAGCAAGCUUCACAGGAGG
**ENV FWD PRIMER (GRNA)**	rGrArU rUrUrA rGrArC rUrArC rCrCrC rArArA rArArC rGrArA rGrGrG rGrArC rUrArA rArArC rGrGrC rUrArU rArCrA rUrUrC rUrUrA rCrUrA rUrUrU rUrArU rUrUrA rA
**CAS13A FLUORO REPORTER**	/5Alex488N/TrUrUrUrUrUrC/3IABkFQ/

Supplementary Table 5. CRISPR/Cas13a Assay

NAME	5'-SEQUENCE-3'
**DNA TEMPLATE**	CAT CGC ACT ACT CAT GAA CTT CAA GCT AAT ACG ACT CAC TAT AGA TGA TTC TGA ATT AGC TGT ATC GTC AAG GCA CTC TTG CCT ACG CCA CAA GCT CCA ACT ACC ACA AGT TTA TAT TCA GTC ATT TTC AGC AGG CCT TCA TCG TAC TGG TGA AAA CAC CGC AGC ATG TCA AGA TCA CAG ATT TTG GGC GGG CCA AAC TGC TGG GTG CGG AAG AGA AAG AAT ACC ATG CAG AAG GAG CAT ATC CAG ACA ACT GTT CAA ACT GAT GGG ACC CAC TCC ATC GAG ATT TCT CTG TAG CTA GAC CAA AAT CAC CTA TTT TTA CTG TGA GGT CTT CAT GAC ATC ATG TTC CTG ATC TCC TTA GAC AAC TAC CTT TCT ACG GAC GTG GGA TTC TGC ACC CTC GTC TGC CCC CTG CAC AAC CAA GAG GTG ACA GCA GAG GCA TTA GGG TCC ATA GTG GGA AAG GTC AGT AAC CAG GCA GAA ACG TAC ATG CTG GGA GCA GAC ACA AAT GTG CTT ATC ACC ATC CCT GAG GTT ATC TGC CAT TGA GCA AGA GGC TTT GGA GTA TTT CAT GAA ACA AAT GAA TGA TGC ACG TCA TGG TGG CTG GAC AAC AAA TAT GGA TTG GAT CTT CCA CAC AAT TAC ATG GGC AGA GGG GAG TTG GGG TGA GGG TGT CTC TCT GTG GCT TTA CCT GAT GAT CAG GGC TTC CAT GAG GAA ATC CAG TTC GTC CTG TTC AGA GCA CCA TTC GCG GCG GAC GAC ACC TGC CGG ACC AGC CAG GGC TCC CCG GCT TTC TAG CCG CCC GAG ATT GCC AAC GGC CTG GAC ACC TTC TCC GGC TTC AAG CTA CGT AAG CTT CAA CTG CAT CCA T
**FORWARD PRIMER**	CGC ACT ACT CAT GAA CTT CAA GC
**REVERSE PRIMER**	GAT GCA GTT GAA GCT TAC GTA GC
**EGFR L858R**	rUrArAr UrUrUr CrUrAr CrUrAr ArGrUr GrUrAr GrArUr GrCrCr CrGrCr CrCrAr ArArAr UrCrU rCrUrGr ArUrC rUrU
**CAS12A/DNASE FQ REPORTER**	/5Alex488N/TT ATT /3IABkFQ/

Supplementary Table 6. CRISPR/Cas12a Assay

NAME	SEQUENCE
**MIR-375**	rUrUrUrGrUrUrCrGrUrUrCrGr GrCrUrCrG rCrGrUrGrA
**LINKER**	TTCACGCGAGCCGAACGAACAAACAACTC/36-FAM/
**PROTECTOR**	/5IABkFQ/TTGTTGTTTGTTCGTTCGGCTCG
**FUEL**	GAGTTGTTTGTTCGTTCGGCTCGC

Supplementary Table 7. Sequences for TRAP assay.

Disease	IVD test	TRAP Compatibility
Acute hepatitis E	anti-HEV IgM	-
Acute hepatitis E	Hepatitis E virus nucleic acid test	Yes
Acute myocardial infarction	hs-cTnI	-
Acute myocardial infarction	hs-cTnT	-
Anatomical pathology	Cytology (cytopathology)	-
Anatomical pathology	Histopathology	-
Anatomical pathology	Immunohistochemistry (IHC)	-
Anatomical pathology	Post-mortem examination	-
Aspergillosis	Aspergillus antigen test	-
Aspergillosis	Aspergillus IgG antibody	-
Blood typing	ABO and RH	-
Cancer	Alpha-fetoprotein (AFP)	-
Cancer	Basic immunohistochemical (IHC) panel for diagnosis of lymphoma	-
Cancer	Basic immunohistochemical (IHC) panel for diagnosis of solid tumours	-
Cancer	BCR-ABL1 and ABL1 transcripts	Yes
Cancer	Epidermal growth factor receptor (EGFR) gene mutation	-
Cancer	erbB-2/ HER-2 overexpression	-
Cancer	Essential flow cytometry panel for leukaemia	-
Cancer	Faecal immunochemical test (FIT)	-
Cancer	Human chorionic gonadotrophin (hCG) (measured as total beta-hCG)	-
Cancer	Lactate dehydrogenase (LDH)	-
Cancer	Oestrogen (ER) and progesterone (PgR) receptors	-
Cancer	Papanicolaou (Pap) smear	-
Cancer	Prostate specific antigen (PSA)	-
Chagas disease	Trypanosoma cruzi IgG antibody	-
Cholera	Vibrio cholerae antigen	-
Clinical chemistry	Alanine aminotransferase (ALT)	-
Clinical chemistry	Albumin	-
Clinical chemistry	Alkaline phosphatase (ALP)	-

Clinical chemistry	Aspartate aminotransferase (AST)	-
Clinical chemistry	Bilirubin	-
Clinical chemistry	Blood pH and gases	-
Clinical chemistry	BUN	-
Clinical chemistry	C-reactive protein (CRP)	-
Clinical chemistry	Creatinine	-
Clinical chemistry	Direct and indirect bilirubin	-
Clinical chemistry	Electrolytes	-
Clinical chemistry	Gamma-glutamyl transferase (GGT)	-
Clinical chemistry	Globulin	-
Clinical chemistry	Glucose	-
Clinical chemistry	Ionized calcium	-
Clinical chemistry	Ketones	-
Clinical chemistry	Lipase or amylase	-
Clinical chemistry	Lipid profile	-
Clinical chemistry	Magnesium	-
Clinical chemistry	Phosphate	-
Clinical chemistry	Procalcitonin	-
Clinical chemistry	Total bilirubin	-
Clinical chemistry	Total calcium	-
Clinical chemistry	Total protein	-
Clinical chemistry	Troponin T/I	-
Clinical chemistry	Uric acid	-
Clinical chemistry	Urinalysis test strips	-
Clinical chemistry	Urine chemistry	-
Clinical chemistry	Whole blood lactate	-
Clinical microbiology	Antimicrobial susceptibility testing	-
Clinical microbiology	Blood culture	-
Clinical microbiology	Culture	-
Clinical microbiology	Genus and species identification of bacteria and fungi	Yes

Clinical microbiology	Staining procedures	-
Clinical Pathology	Body fluid microscopy	-
Clinical Pathology	Urine microscopy	-
Congenital adrenal hyperplasia	17-OHP	-
Coronavirus disease (COVID-19)	SARS CoV-2 nucleic acid test	Yes
Diabetes mellitus	Haemoglobin A1c (HbA1c)	-
Disorders of the parathyroids or parathyroid hormone system	PTH	-
Endocrine disorders	Cortisol (total)	-
Endocrine disorders	Estradiol	-
Endocrine disorders	Follicle- stimulating hormone (FSH)	-
Endocrine disorders	Luteinizing hormone (LH)	-
Endocrine disorders	Progesterone	-
Endocrine disorders	Prolactin	-
Endocrine disorders	Thyroid-stimulating hormone (TSH)	-
Haematology	Basic cerebrospinal fluid (CSF) Profile	-
Haematology	Blood crossmatching	-
Haematology	Complete blood count (CBC) Automated	-
Haematology	D-Dimer	-
Haematology	Direct antiglobulin test (DAT)/ Direct Coombs test	-
Haematology	Erythrocyte sedimentation rate (ESR)	-
Haematology	Fibrinogen	-
Haematology	Haematocrit (Hct)	-
Haematology	Haemoglobin (Hb)	-
Haematology	Indirect antiglobulin test (IAT)/Indirect Coombs test	-
Haematology	Iron studies	-
Haematology	Partial thromboplastin time (PTT)/ Activated partial thromboplastin time (APTT)	-
Haematology	Peripheral blood film examination	-
Haematology	Platelet count	-
Haematology	Prothrombin time and international normalized ratio (PT/INR)	-
Haematology	Sickle cell testing	-

Haematology	White blood cell count	-
Haemolytic disease of fetus or newborn	ABO and RH	-
Haemolytic disease of fetus or newborn	Kleihauer-Betke acid-elution test	-
Hepatitis B virus (HBV) infection	Hepatitis B e antigen (HBeAg)	-
Hepatitis B virus (HBV) infection	Hepatitis B IgM core antibody (IgM anti-HBc)	-
Hepatitis B virus (HBV) infection	Hepatitis B quantitative nucleic acid	Yes
Hepatitis B virus (HBV) infection	Hepatitis B surface antibody (anti-HBs)	-
Hepatitis B virus (HBV) infection	Hepatitis B surface antigen (HBsAg)	-
Hepatitis C virus (HCV) infection	Hepatitis C antibodies + HCV core antigen (HCV cAg)	-
Hepatitis C virus (HCV) infection	Hepatitis C antibody (Anti-HCV Ab)	-
Hepatitis C virus (HCV) infection	Hepatitis C core antigen (HCVcAg)	-
Hepatitis C virus (HCV) infection	Hepatitis C qualitative or quantitative nucleic acid	Yes
HIV infection	CD4 cell enumeration	-
HIV infection	Combined HIV antibody/p24 antigen (anti-HIV/p24 Ag)	-
HIV infection	Cryptococcal antigen	-
HIV infection	Histoplasma antigen	-
HIV infection	HIV 1/2 antibody (anti-HIV Ab)	-
HIV infection	HIV 1/2 antibody + p24 antigen	-
HIV infection	HIV qualitative nucleic acid test	-
HIV infection	HIV quantitative nucleic acid test	Yes
Human papillomavirus (HPV) Infection	HPV nucleic acid test	Yes
Influenza	Influenza A and B antigen	-
Influenza	Influenza A and B nucleic acid	Yes
Malaria	Antibodies to Plasmodium spp.	-
Malaria	Glucose-6- phosphate dehydrogenase activity (G6PD)	-
Malaria	Plasmodium spp.	-
Malaria	Plasmodium spp. antigens	-
Neglected tropical diseases	Dengue virus IgM antibody	-
Neglected tropical diseases	Dengue virus NS1 antigen	-
Neglected tropical diseases	Dengue virus qualitative nucleic acid	Yes

Neglected tropical diseases	Kato-Katz	-
Neglected tropical diseases	Visceral leishmaniasis direct agglutination test	-
Other transfusion-transmitted organisms	To screen for other blood-transmitted microorganisms	-
Pneumocystis pneumonia	Pneumocystis jirovecii nucleic acid test	Yes
Pregnancy testing	Human chorionic gonadotropin (hCG)	-
Primary Immunodeficiencies	Immunoglobulin levels (IgG, IgA, IgM)	-
Primary Immunodeficiencies	Lymphocyte subtype enumeration	-
Sexually transmitted infections	Chlamydia trachomatis (CT) and Neisseria gonorrhoeae (NG) Qualitative nucleic acid	Yes
Sexually transmitted infections	Treponema pallidum + HIV-1/2 antibodies;HIV 1/2 + Treponema pallidum antibodies	-
Sexually transmitted infections	Venereal disease research laboratory (VDRL)	-
Streptococcal pharyngitis	Group A Streptococcus antigen	-
Syphilis	Antibodies to T. pallidum	-
Syphilis	Combined antibodies to T. pallidum and HIV-1/2	-
Syphilis	Rapid plasma reagin (RPR)	-
Syphilis	T. pallidum haemagglutination (TPHA)	-
Syphilis	T. pallidum particle agglutination (TPPA)	-
Syphilis	Treponema pallidum antibodies	-
Tuberculosis (TB)	Lipoarabinomannan (LAM) antigen	-
Tuberculosis (TB)	M. tuberculosis DNA	Yes
Tuberculosis (TB)	M. tuberculosis DNA mutations associated with resistance	Yes
Tuberculosis (TB)	M. tuberculosis DNA resistance mutations	Yes
Tuberculosis (TB)	M. tuberculosis drug susceptibility testing	-
Tuberculosis (TB)	M. tuberculosis Immune response by Interferon gamma release assay (IGRA)	-
Tuberculosis (TB)	Mycobacterium tuberculosis bacteria	-
Tuberculosis (TB)	Tuberculin skin (Mantoux) test (TST)	-
Vaccine-preventable diseases	Measles IgM antibody	-
Vaccine-preventable diseases	Measles IgM antibody	-
Vaccine-preventable diseases	Measles nucleic acid test	Yes
Vaccine-preventable diseases	Rubella IgG antibody	-
Vaccine-preventable diseases	Rubella IgM antibody	-

Visceral leishmaniasis (kala-azar)	rK39 antigen	-
Zika virus infection	Zika virus IgM antibodies	-
Zika virus infection	Zika virus qualitative nucleic acid test	Yes

Supplementary Table 8. WHO Essential In Vitro Diagnostics List (EDL) and TRAP compatibility.

	ZIKV			MSSA			HIV			L858R EGFR			miR-375-3p	
	RT-LAMP			RPA			CRISPR/Cas13			CRISPR/Cas12			TRAP	
$copies/\mu L$	log(RFU)	CV (%)	$copies/\mu L$	log(RFU)	CV (%)	nM	log(RFU)	CV (%)	nM	log(RFU)	CV (%)	pM	log(RFU)	CV (%)
$1x10^6$	3.74 ± 0.01	0.21	$1x10^4$	2.79 ± 0.03	1.19	$1x10^1$	3.16 ± 0.02	0.69	$1x10^1$	3.67 ± 0.10	2.73	$5x10^3$	2.95 ± 0.13	4.15
$1x10^5$	3.70 ± 0.01	0.32	$1x10^3$	2.69 ± 0.01	0.23	$1x10^0$	2.30 ± 0.05	2.22	$1x10^0$	3.27 ± 0.05	1.36	$5x10^2$	1.76 ± 0.16	8.87
$1x10^4$	3.61 ± 0.04	1.11	$1x10^2$	2.43 ± 0.04	1.73	$1x10^{-1}$	1.19 ± 0.03	3.26	$1x10^{-1}$	2.16 ± 0.05	2.32	$5x10^1$	-*	-*
$1x10^3$	3.57 ± 0.04	1.06	$1x10^1$	2.32 ± 0.01	0.12	$1x10^{-2}$	0.76 ± 0.09	12.46	$1x10^{-2}$	1.39 ± 0.05	3.87	$5x10^0$	-*	-*
0	3.42 ± 0.04	1.13	0	2.33 ± 0.01	0.47	0	0.70 ± 0.09	13.43	0	0.28 ± 0.49	173.21	0	-*	-*
Overall CV (%)		0.766			0.748			6.41			36.70			6.51

* log(RFU) is undefined due to negative Δ fluorescence signal
CV coefficient of variation

Supplementary Table 9. Intra-assay variability of the VPodDuo. For each assay, three replicates were tested individually using single master reagent mix.

Supplementary Methods

S1. Excitation and emission sequence

As illustrated in **Fig. 2d**, the measurement sequence has apparent delay between each excitation light initiation and emission light collection. This measurement delay arises from the operating principle of the photodiode sensor, which continuously integrates ambient light over a predefined integration time interval without providing the access to explicit control over the exact start time of a set integration duration. Moreover, when queried, the sensor does not return an instantaneous or in-progress reading; rather, it outputs the most recently completed integration result. Given this behavior, the relationship between the time profile of the fluorescence excitation signal and the integration behavior of the sensor can be conceptually described using a convolution formalism:

$$I_{measured}(t) = \left(I_{fluor} \circledast H_{sensor}\right)(t) = \int_{-\infty}^{\infty} I_{fluor}(\tau) H_{sensor}(t-\tau)\, d\tau$$

$$where$$

$$I_{fluor}(t) = \begin{cases} f(t), & 0 \le t \le A \\ 0, & otherwise \end{cases},$$

$$H_{sensor}(t) = \begin{cases} S, & 0 \le t \le B \\ 0, & otherwise \end{cases} \qquad (eq. 1)$$

Eq. 1 describes the convolution operation $\circledast$, where $I_{fluor}(t)$ represents the fluorescence intensity induced by laser excitation, modeled as a rectangular pulse with width A, corresponding to the laser excitation duration. $f(t)$ represents the fluorescence intensity over time as a function of assay's amplification kinetics where it can take forms of linear or exponential, depending on the type of assay. $H_{sensor}(t)$ denotes the sensor integration response function, modeled as a rectangular pulse with width B, representing the sensor integration time, and scaled by an amplitude factor S determined by the photodiode sensor's static response, including analog or digital gain, photosensitivity, and quantum efficiency. For simplicity, $f(t)$ and S can take a form of unit rectangular function under the assumptions of instantaneous and stable fluorescence output (i.e., unaffected by photobleaching, photoblinking, and photochemical decay), and constant fluorophore concentration. In the case of the sensor integrating the emission intensity before laser illumination, the initial measured signal may include partial or no fluorescence. The emission intensity is fully captured only when the sensor integration starts after an interval B has passed since the start of laser illumination, ensuring the integration window fully overlaps with the fluorescence signal. This indicates that, to ensure consistent and reliable capture of fluorescence intensity, the excitation duration must be at least twice the predefined integration time, guaranteeing full overlap between the fluorescence signal and the sensor's integration window, regardless of alignment. To account for laser intensity fluctuations, a total delay of two seconds is introduced before taking the fluorescence measurement. Furthermore, to minimize the

photobleaching of fluorescence reporters, each measurement was taken at one-minute intervals for a total duration of 10 minute testing time.

S2. Reverse transcription loop mediated isothermal amplification (RT-LAMP) for ZIKV detection

Gamma-irradiated and inactivated Zika virus strain PRVABC59 (NR-50547) was sourced from BEI Resources. The stock was aliquoted, stored at −80°C, and diluted with nuclease-free water to achieve the desired concentrations. The RT-LAMP assay specifically targeted the RNA sequence within the NS2a gene for amplification [1]. All sequences are shown in **Supplementary Table 3.**

The reaction mixture included the following components: 4 mM $MgSO_4$ and 1× isothermal amplification buffer (both from New England Biolabs), 0.29 M betaine (Sigma-Aldrich), 1.025 mM of each dNTP, 0.3 U/μL WarmStart Reverse Transcriptase, 0.47 U/μL Bst 2.0 WarmStart DNA Polymerase (New England Biolabs), 1 mg/mL BSA (New England Biolabs), and 0.735× EvaGreen dye (Biotium; 0.92 μM). Primer concentrations were set at 0.2 μM for F3 and B3, 1.6 μM for FIP and BIP, and 0.8 μM for LoopB. The final reaction volume of 16 μL comprised 2 μL of RNA template and 14 μL of the RT-LAMP master mix. Reactions were assembled in 0.2 mL PCR tubes.

The RT-LAMP reactions were conducted at 65°C for 10 minutes using a QuantStudio™ 3 Real-Time PCR System (Applied Biosciences). Fluorescence signals were recorded at one-minute intervals to generate amplification curves. Following this, the PCR tubes were removed and analyzed using the VPodDuo system. Each experiment was conducted in triplicate, and the fluorescence intensity observed at the 10-minute mark was log-transformed and compared with the endpoint data obtained from VPodDuo at the same time point.

S3. Recombinase polymerase amplification (RPA) for MSSA detection

Genomic DNA from MSSA strain Seattle 1945 (25923D-5) was obtained from ATCC (Manassas, VA, USA). After aliquoting, the stock was stored at −80°C and diluted in nuclease-free water to achieve the required working concentrations. Primers targeting the vicK gene of MSSA were selected from the literature [2]. All sequences are shown in **Supplementary Table 4.**

The RPA assay utilized the TwistAmp exo kit (TwistDx, United Kingdom) with slight modifications to the manufacturer's protocol. The reaction setup included 2.1 μL of forward and reverse primers (10 μM each), 0.6 μL of an exo probe (10 μM), 11.2 μL of nuclease-free water, and 29.5 μL of rehydration buffer. This mixture was manually prepared in a 1.5-mL tube, transferred to the lyophilized RPA pellet for resuspension, and combined with 2 μL of the DNA sample.

Before loading the samples, 2.5 μL of 280 mM magnesium acetate (MgOAc) was added to the tube lid and centrifuged to ensure proper mixing. The total volume of the reaction mix was 50 μL. RPA reactions were carried out in 0.2-mL PCR tubes at 37°C for 10 minutes using the QuantStudio™ 3 Real-Time PCR System (Applied Biosciences). Fluorescence was tracked at one-

minute intervals to create amplification curves. Afterward, the PCR tubes were removed for analysis using the VPodDuo system. All experiments were performed in triplicate, and the log-transformed fluorescence data were compared to endpoint measurements obtained through VPodDuo.

S4. CRISPR/Cas13 for HIV detection

All sequences are listed in Supplementary Table 5, with sequence sources referenced to Nouri et al.[3]. A segment of the HIV-1 genome was purchased from IDT and transcribed into RNA using the HiScribe T7 High Yield RNA Synthesis Kit (New England Biolabs), followed by purification with the Monarch RNA Cleanup Kit (New England Biolabs). RNA concentration was determined using a Nanodrop One Spectrophotometer (Thermo Scientific). Lyophilized Cas13a enzyme (MCLabs) was reconstituted in 20 mM HEPES buffer with 1 mM $MgCl_2$, pH 6.8. Guide RNA (gRNA) was designed to target the ENV gene, purchased in lyophilized form (IDT), and resuspended in pH 8 IDTE buffer to 100 µM concentration. The Cas13a enzyme and gRNA were combined in a 1:1 molar ratio and incubated at room temperature for 30 minutes to form the Cas13a:gRNA complex (250 nM each) in 4 µL for each reaction. Fluoro-reporter (IDT) was resuspended in IDTE buffer (pH 7.5) to a 100 µM stock solution. The 100 µL final reaction mixture contained 10 nM Cas13a:gRNA complex, 1 µM fluorogenic reporter, 1 U/mL Murine RNase Inhibitor and serial dilutions of the target RNA template from 10 nM to 10 pM. Reactions proceeded for 10 minutes while fluorescence was measured at one minute interval. The both VPodDuo and micro-plate reader measurements were taken at 37°C.

This assay is based on the SHERLOCK platform developed by Gootenberg *et al.* [3], where Cas13a is activated upon binding a specific target nucleic acid, triggering cleavage of a fluorogenic reporter. The gRNA, designed to recognize the HIV-1 ENV gene, guides Cas13a to its target. Upon activation, Cas13a indiscriminately cleaves nearby reporter probes, releasing the quenched fluorophore and enabling fluorescence detection.

S5. CRISPR/Cas12a for L858R point-mutated EGFR detection

All oligonucleotide sequences (crRNA, primers, target DNA, and fluorophore-quencher [FQ] reporter) listed in Supplementary Table 6 were purchased from Integrated DNA Technologies (IDT) in lyophilized form. All lyophilized oligonucleotide were suspended in IDTE buffer (pH 7.5) containing Murine RNase Inhibitor (1 U/mL). The target DNA were amplified via PCR with Q5 polymerase (New England Biolabs) using forward and reverse primers. Following amplification, the PCR products were purified using the Monarch PCR and DNA Cleanup Kit (New England Biolabs), and their concentrations were determined using a Nanodrop One Spectrophotometer (Thermo Scientific). Cas12a (LbCas12a) was purchased from New England Biolabs and diluted in EnGen Lba Cas12a Diluent. For complex formation, Cas12a (100 nM) and the crRNA (125 nM) were combined at a 1:1.25 ratio in 1X NEBuffer 2.1 to a total volume of 50 µL and incubated at 37 °C for 30 minutes. Seperately, a DNA/FQ reporter master mix was then prepared containing 1

μM FQ reporter and target DNA in 50 μL reaction volume. The DNA/FQ master mix was added to the Cas12a–crRNA complex to reach a final reaction volume of 100 μL. The final concentrations in the reaction are 50 nM Cas12a, 62.5 nM crRNA, and 500 nM FQ reporter. Target DNA concentrations ranged from 10 nM to 10 pM in the final reaction mixture. Fluorescence readings were measured in both a microplate reader and VPodDuo at 37°C for 10 minutes with measurement taken at one minute interval.

The CRISPR-based detection assay was based on the DETECTR assay, developed by Chen *et al.* [4]. DETECTR employs Cas12a (also referred to as "LbCas12a") and a target-specific guide RNA (crRNA) to recognize the DNA template. Upon target binding, Cas12a undergoes an allosteric transition, activating its collateral cleavage activity, which in turn cleaves any single-stranded DNA (ssDNA) bearing a fluorescent/quencher pair, thus generating a quantifiable fluorescent signal.

S6. Target recycling amplification process (TRAP)

TRAP was demonstrated for the detection of miR-375-3p. Sequences were designed using previously tested toehold-mediated strand displacement assays demonstrated by Wang *et al.* [5]. The linker strand was modified with a 3' FAM fluorophore, and the protector strand was modified with a 5' Iowa Black Hole Quencher. The linker and protector strands were pre-annealed at a ratio of 1:2 of linker to protector, with the protector in excess, by heating to 80°C then slowly cooling to room temperature, in a 1xTE, 12.5 mM MgCl2 (Invitrogen, #11568976, #AM9530G), buffer. This effectively quenches the fluorophore. The target microRNA reacts with a toehold region initially exposed on the linker substrate, which activates a toehold-mediated strand displacement reaction, removing the protector, and activating the fluorophore. This simultaneously exposes a secondary toehold on the linker strand, which can then react with the fuel sequence which displaces the target microRNA back into solution to continue to react. The toehold lengths were slightly increased to create faster amplification for bulk fluorescence detection, with the initial toehold composed of 6 nucleotide bases, and the secondary sequestered toehold composed of 8 nucleotide bases. The fuel strand had an added 1 nucleotide overhang for the initial toehold to further increase reactivity.

The TRAP reaction for miR-375 was performed for a negative control and four tested concentrations (5 nM, 500 pM, 50 pM, 5 pM, and 0 nM). The reaction was performed in 0.5xPBS (Fischer, 21040CV), 0.5xTE (Invitrogen, #11568976), and 5 mM MgCl2 buffer (Invitrogen, #AM9530G). Annealed linker-protector duplex and fuel sequences were added to 0.7 mL tube at a final concentration of 250 nM and 7.5 μM, respectively, then the microRNA was added to its final tested target concentration, all at a total reaction volume of 100 μL. The TRAP reagents were allowed to react at room temperature for 10 minutes and were tested either with VPodDuo system or a commercial QubitTM fluorometer at one-minute intervals to show change in relative fluorescence. All sequences were ordered from Integrated DNA Technologies (Coralville, IA) with HPLC purification and shown in **Supplementary Table 7.**

References

[1] A. Calvert, B. Biggerstaff, N. Tanner *et al.*, "Rapid colorimetric detection of Zika virus from serum and urine specimens by reverse transcription loop-mediated isothermal amplification (RT-LAMP)," *PLOS ONE,* vol. 12, pp. e0185340, 2017/09/25/, 2017.

[2] M. Schulz, S. Calabrese, F. Hausladen *et al.*, "Point-of-care testing system for digital single cell detection of MRSA directly from nasal swabs," *Lab on a Chip,* vol. 20, no. 14, pp. 2549-2561, 2020, 2020.

[3] J. S. Gootenberg, O. O. Abudayyeh, J. W. Lee *et al.*, "Nucleic acid detection with CRISPR-Cas13a/C2c2," *Science,* vol. 356, no. 6336, pp. 438-442, 2017/04/28/, 2017.

[4] J. S. Chen, E. Ma, L. B. Harrington *et al.*, "CRISPR-Cas12a target binding unleashes indiscriminate single-stranded DNase activity," *Science,* vol. 360, no. 6387, pp. 436-439, 2018/04/27/, 2018.

[5] X. Wang, S. Shepherd, N. Li *et al.*, "A Target Recycling Amplification Process for the Digital Detection of Exosomal MicroRNAs through Photonic Resonator Absorption Microscopy," *Angewandte Chemie International Edition,* vol. 62, no. 16, pp. e202217932, 2023/04/11/, 2023.
